# Biochemical Characterization of a Novel Monospecific Endo-β-1,4-Glucanase Belonging to GH Family 5 From a Rhizosphere Metagenomic Library

**DOI:** 10.3389/fmicb.2019.01342

**Published:** 2019-06-14

**Authors:** Anna Wierzbicka-Woś, Ruth Henneberger, Ramón Alberto Batista-García, Liliana Martínez-Ávila, Stephen A. Jackson, Jonathan Kennedy, Alan D. W. Dobson

**Affiliations:** ^1^Environmental Research Institute, University College Cork, Cork, Ireland; ^2^Department of Microbiology, Faculty of Biology, University of Szczecin, Szczecin, Poland; ^3^Institute for Molecular Health Sciences, ETH Zürich, Zurich, Switzerland; ^4^Centro de Investigación en Dinámica Celular, Instituto de Investigación en Ciencias Básicas y Aplicadas, Universidad Autónoma del Estado de Morelos, Cuernavaca, Mexico; ^5^School of Microbiology, University College Cork, Cork, Ireland; ^6^Centre for Process Innovation, Wilton, United Kingdom

**Keywords:** functional metagenomic, cellulases, glycosyl hydrolase family 5, endoglucanase, psychrotolerant cellulolytic enzyme

## Abstract

Cellulases have a broad range of different industrial applications, ranging from food and beverages to pulp and paper and the biofuels area. Here a metagenomics based strategy was used to identify the cellulolytic enzyme CelRH5 from the rhizosphere. CelRH5 is a novel monospecific endo-β-1,4-glucanase belonging to the glycosyl hydrolase family 5 (GH5). Structural based modeling analysis indicated that CelRH5 is related to endo-β-1,4-glucanases derived from thermophilic microorganisms such as *Thermotoga maritima*, *Fervidobacterium nodosum*, and *Ruminiclostridium thermocellum* sharing 30-40% amino acid sequence identity. The molecular weight of the enzyme was determined as 40.5 kDa. Biochemical analyses revealed that the enzyme displayed good activity with soluble forms of cellulose as a substrate such as ostazin brilliant red hydroxyethyl cellulose (OBR-HEC), carboxymethylcellulose (CMC), hydroxyethyl cellulose (HEC), and insoluble azurine cross-linked hydroxyethylcellulose (AZCL-HEC). The enzyme shows highest enzymatic activity at pH 6.5 with high pH tolerance, remaining stable in the pH range 4.5–8.5. Highest activity was observed at 40°C, but CelRH5 is psychrotolerant being active and stable at temperatures below 30°C. The presence of the final products of cellulose hydrolysis (glucose and cellobiose) or metal ions such as Na^+^, K^+^, Li^+^, and Mg^2+^, as well as ethylenediaminetetraacetic acid (EDTA), urea, dithiothreitol (DTT), dimethyl sulfoxide (DMSO), 2-mercaptoethanol (2-ME) or glycerol, did not have a marked effect on CelRH5 activity. However, the enzyme is quite sensitive to the presence of 10 mM ions Zn^2+^, Ni^2+^, Co^2+^, Fe^3+^ and reagents such as 1 M guanidine HCl, 0.1% sodium dodecyl sulfate (SDS) and 20% ethanol. Given that it is psychrotolerant and retains activity in the presence of final cellulose degradation products, metal ions and various reagents, which are common in many technological processes; CelRH5 may be potential suitability for a variety of different biotechnological applications.

## Introduction

Cellulases are extensively used in a variety of different industrial sectors including the healthcare, food, beverage, textile, pulp and paper sectors, as well as more recently in the biofuels sector (Sukumaran et al., 2005; Zhang and Kim, 2010; Kuhad et al., 2011; Bashir et al., 2014; Lambertz et al., 2014; Meneses et al., 2016). With the ever increasing demand on our rapidly depleting fossil fuel supply, the production of second generation biofuels from abundant lignocellulosic biomass sources such as agricultural and forestry wastes has become attractive as a sustainable and alternative option (Tiwari et al., 2018). Enzymatic lignocellulose hydrolysis using cellulases is one of the key steps in biofuel production from lignocellulosic biomass. Due to this, the global market for biofuel enzymes continues to increase and will reach $1.0 billion by 2020, with the European market alone set to be worth $325.2 million in the same year^1^.

Cellulases are produced not only by plants and some insects feeding on cellulose, but also by a broad group of microorganisms including fungi, bacteria (Pérez et al., 2002; Sukumaran et al., 2005; Zhang and Kim, 2010; Kuhad et al., 2011; Lambertz et al., 2014; Meneses et al., 2016) and archaea (Graham et al., 2011). They are glycosyl hydrolases (GH) (including β-glucosidase, endo- and exo-glucanase) that catalyze the degradation of cellulose into glucose and other fermentable sugars. Exoglucanases (EC 3.2.1.91 and 3.2.1.176) progressively act on reducing and non-reducing ends of the cellulose to release cellobiose moieties. Endoglucanases (EC 3.2.1.4) randomly cleave internal β-1,4-glucan linkages, while β-glucosidases (EC 3.2.1.21) degrade di-saccharides to release glucose units. These enzymes are typically produced in prokaryotes as multi-enzymes complexes called cellulosomes (Pakarinen et al., 2014; Sharma et al., 2016).

Given the aforementioned demand for biofuel, there is an ongoing interest in the discovery of novel cellulases and enzymes which decompose plant biomass. In particular, cellulases that possess high catalytic activity on insoluble substrates, coupled with higher tolerance to end-product inhibition, are attractive from an industrial perspective (Ryu and Karim, 2011). While cellulases with industrially relevant characteristics have previously been obtained from cultured microorganisms, there is still a need for cellulases that are resistant to biomass pre-treatment conditions, such as high temperature and acid/alkaline conditions, among others (Horn et al., 2012; Mori et al., 2014; Vester et al., 2014). In this context, culture-independent metagenomics based approaches are increasingly being employed to discover novel cellulases with new biochemical properties, using both sequence and functional based approaches (Xing et al., 2012; Garg et al., 2016; Yang et al., 2016). Functional approaches do not depend on the availability of prior sequence information to detect cellulases and therefore there is greater potential to discover genetic novelty. These approaches have resulted in the identification of cellulases from compost, rumen, soil, and decaying wood metagenomes, as these environments are rich in microbial consortia which efficiently decompose plant biomass (Allgaier et al., 2010; Li et al., 2011; Ferrer et al., 2012; Nacke et al., 2012). Also, the rhizosphere, a narrow zone surrounding and influenced by plant roots, is considered to be one of the most complex ecosystems on Earth. Moreover, the rhizosphere microbiome may reach cell densities much greater than the number of plant cells and when comparing the numbers of genes present, microbial genes far outnumber the plant genes present. Furthermore, glycosyl hydrolases which decompose cellulose are very abundant in soils including in the rhizosphere (Mendes et al., 2013; Berlemont and Martiny, 2016). With this in mind, we set out to assess the potential to identify novel cellulases from a metagenomic library obtained from rhizosphere soil samples collected from a non-fertilized grassland, using a functional based approach. Following screening of approximately 15,600 clones, three positive clones were identified on LB (Luria Bertani) medium supplemented with ostazin brilliant red H-3B hydroxyethyl cellulose (OBR-HEC). One of these clone, named as RH5_TO-NF021-E23, displayed the best activity and the enzyme was subsequently biochemically characterized following heterologous expression in *Escherichia coli*. The recombinant CelRH5 was found to be a novel monospecific endo-β-1,4-glucanase belonging to GH family 5 (GH5). CelRH5 displayed enzymatic activity in the presence of OBR-HEC, carboxymethylcellulose (CMC), hydroxyethyl cellulose (HEC), azurine-cross-linked hydroxyethyl cellulose (AZCL-HEC), and was active over a broad pH range (4.5–8.5), with an optimal activity at pH 6.5. The enzyme also showed tolerance to low temperatures being functional and stable below 30°C and activity was not inhibited by either glucose or cellobiose. Cellulase activity was not affected by the addition of various metal ions such as Na^+^, K^+^, Li^+^, and Mg^2+^. In addition, ethylenediaminetetraacetic acid (EDTA), urea, dithiothreitol (DTT), dimethyl sulfoxide (DMSO), 2-mercaptoethanol (2-ME) and glycerol did not affect the activity of the recombinant CelRH5 enzyme.

## Materials and Methods

### Sampling and Metagenomic Library Construction

Rhizosphere samples (1–5 cm below the surface) were collected from non-fertilized grassland surrounding an organic field trial site at the Teagasc Oak Park (Carlow, Ireland, 52.8657° N, 6.9129° W). Soil samples (TO-NF) were collected and handled with sterile, DNA-free tools (treated with 5% sodium hypochlorite for 30 min prior to washing and autoclaving) and stored in sterile, DNA-free plastic containers. Samples were transported to the laboratory on ice, aseptically fractionated and stored at 4°C until further processing.

High molecular weight (HMW) metagenomic DNA was extracted, purified and a metagenomic library was constructed using the CopyControl^TM^ Fosmid Library Production Kit pCC1FOS (Epicentre Biotechnologies) as previously described (O’Mahony et al., 2015). Approximately 45,000 clones were picked with the Genetix Qpix2 XT robotic system (Genetix, New Milton, United Kingdom) into 384-well plates containing LB media (1% peptone, 1% NaCl, 0.5% yeast extract; pH 7.0) enriched with: 6.3 g/L K_2_HPO_4_, 1.8 g/L KH_2_PO_4_, 0.5 g/L sodium citrate dihydrate, 0.09 g/L MgSO_4_ × 7H_2_O, 6% (v/v) glycerol. After the incubation at 37°C for 18–20 h, the library was stored at $-<cps:it>80</cps:it{>}^{\circ }$C.

To determine the average insert size, twelve recombinant fosmid clones were randomly picked, used in *Not*I digestion reactions and analyzed by Pulsed-Field Gel Electrophoresis (PFGE) with the following conditions: 1% agarose in 0.5% Tris-Borate-EDTA (TBE) buffer, 6 V/cm, 1–25 s switch time, 120° angle, 11.5 h and 14°C. Six randomly selected fosmid clones were end-sequenced using the pCC1^TM^/pEpiFOS^TM^ forward and reverse primer (Epicentre Biotechnologies). Sequencing was performed by GATC Biotech (Konstanz, Germany) and sequences were subjected to comparison with public databases using BLASTn algorithms^2^.

### Screening of Metagenomic Library Clones for Cellulase Activity

Recombinant fosmid clones were tested for endoglucanase activity on LB agar plates supplemented with OBR-HEC (supplied by the Department of Chemistry, Slovak Academy of Science, Bratislava, Slovakia), a water-soluble cellulose with approximately 12.5% covalently bound dye. A 1% (w/v) stock solution of OBR-HEC was prepared by stirring 1 g of OBR-HEC in 100 mL of Milli-Q H_2_O overnight in the dark, followed by autoclaving for 15 min. This solution was combined with freshly autoclaved LB agar to a final concentration of 0.1% OBR-HEC and 100 mL of this mixture was then poured into 20 cm × 20 cm trays that contained 200 mL of solidified and cooled LB agar [containing 12.5 μg/mL chloramphenicol and 0.01% (w/v) arabinose].

Approximately 15,600 fosmid clones were replicated into fresh LB medium supplemented with 12.5 μg/mL chloramphenicol and incubated at 37°C overnight. The clones were replicated onto the prepared OBR-HEC agar trays, using the QPix robotic system (Molecular Devices). Plates were incubated at 37°C overnight, followed by further incubation at 25°C for 4–5 days to enable detection of cellulolytic activity, i.e., zones of clearing around the colonies. *E. coli* EPI300^TM^ T1^®^ clones containing pCC1FOS with cloned fosmid control DNA (Epicentre, 36 kb fragment of human DNA), were used as a negative control.

### Sequencing and Bioinformatic Analyses

Recombinant fosmid RH5_TO-NF021-E23 displayed cellulolytic activity and was sequenced by Roche 454 pyrosequencing. A contiguous sequence of approximately 41 kb (partially including the fosmid sequence) was re-assembled by the University of Liverpool, Center for Genomic Research (United Kingdom). The sequence was analyzed for the presence of presumable open-reading frames (ORFs) using FGENESB – Bacterial Operon and Gene Prediction Program (Softberry, Goteborg, Sweden) and the MetaGene program (Noguchi et al., 2006). Nucleotide and amino acid sequences of identified ORFs were then screened using BLAST searches to establish their similarity to other sequences deposited in databases.

The amino acid sequence of the metagenome-derived cellulase CelRH5 was determined using the EMBOSS Transeq application (Rice et al., 2000; McWilliam et al., 2013; Li et al., 2015). To establish the most related nucleotide and amino acid sequences for the *celRH5* gene, BLASTn, BLASTp, and PSI-BLATS analysis were performed (see text footnote 2). The theoretical molecular weight (M_w_) and isoelectric point (pI) were estimated using the ExPASy ProtParam tool Compute MW/pI (Gasteiger et al., 2005). The prediction of functional and structural domains, catalytic sites and signal sequences was performed with applications such as Conserved Domain Database (CDD) (Marchler-Bauer et al., 2015), ScanProsite (De Castro et al., 2006), and SignalP 4.1 (Petersen et al., 2011). A prediction of the potential subcellular localization of CelRH5 in gram-negative bacteria was investigated using TargetP 1.1 (Emanuelsson et al., 2007). Phylogenetic analysis was performed using MEGA 6.0 (Tamura et al., 2013) to establish the phylogenetic relationship with other GH 5 members, with the amino acid sequences of the most related proteins with CelRH5 being used in the phylogeny construction. In addition, other putative endoglucanases and cellulases recovered from the Protein Data Bank (PDB) (Berman et al., 2000), and previously characterized metagenome derived GH5 enzymes, were also included. Sequence alignment was prepared using ClustalW and the phylogenetic tree was constructed using the Maximum Likelihood Method and LG+G model with bootstrap 1000.

### CelRH5 Modeling

To predict the structure and biological function of the CelRH5 protein, based on known proteins deposited in PDB, the CelRH5 amino acid sequence without the signal peptide was submitted to the automated comparative protein modeling iTASSER server (Roy et al., 2010) and a 3D model was obtained. The 3D superposition between CelRH5 and some structural neighbors (PDBs 3MMU, 1CEO, 4YHE, 3NCO, and 3RJX) was prepared using the VMD software (Humphrey et al., 1996).

### Cloning of the *celRH5* Gene in *E. coli*

The following primers were used to amplify the *celRH5* gene and the signal peptide sequence, RH5fl*Pci*I (5′-AAAAC **ATGT ACCCATCAAAAGCGACTGAAAGGC**-3′) and RH5r*Xho*I (5′-AAA***CTCGAG*CAGTGCTCGCCTGATCGGC**-3′) from the fosmid RH5_TO-NF-020-E23. Both primers contained recognition sites for endonucleases *Pci*I and *Xho*I, respectively (underlined) and were designed to facilitate cloning into the expression vector pBAD/*Myc*-HisA (Invitrogen) under control of the arabinose inducible promoter P_BAD_. Two final codons in the gene sequence were modified by replacing the sequence 5′-CTGGAT sequence with 5′-*CTCGAG* (*italic*) to generate a *Xho*I restriction site, resulting in a silent mutation without changing the amino acid endoglucanase sequence, and the terminal stop codon was also removed to allow C-terminal fusion with the 6xHis-tag region of the vector. The PCR reaction used Dream *Taq* Polymerase (Thermo Scientific) under the following conditions: 95°C for 2 min; 34 cycles of denaturation at 95°C for 30 s, primers hybridization at 63°C for 30 s, primers elongation at 72°C for 1 min; and 72°C for 5 min.

The PCR amplified *celRH5* gene was cloned into the pBAD/*Myc*-HisA vector following double digestion with *Nco*I and *Sal*I and the recombinant plasmid was transformed into *E. coli* TOP10 (Invitrogen) cells, plated onto LB agar medium supplemented with L-arabinose (0.02% final concentration), carbenicillin (100 μg/mL final concentration) and OBR-HEC as a chromogenic substrate (0.1% final concentration), and cultivated at 37°C overnight. Recombinant *E. coli* TOP10/pBAD/*celRH5* clones displaying cellulase activity were picked and transferred onto fresh selective LB agar plates. The inserted fragment in the recombinant plasmid was also sequenced by GATC Biotech using primers pBAD forward (5′-ATGCCATAGCATTTTTATCC-3′) and pBAD reverse (5′-GATTTAATCTGTATCAGG-3′) to confirm the sequence of the *celRH5* gene in the pBAD/*Myc*-HisA vector.

### Production of Recombinant Enzyme CelRH5 in *E. coli*

To obtain the highest production yield of active CelRH5 enzyme while minimizing the amount of inactive fraction as inclusion bodies, the following procedure was developed. An *E. coli* TOP10/pBAD/*celRH5* overnight culture was used to inoculate 150 mL of LB medium containing carbenicillin (0.1 mg/mL). Additional spiral coils were placed in the medium providing appropriate aeration. Cultures were grown shaking (180 rpm) at 37°C until OD_600_ reached 0.55. Expression was induced with L-arabinose at a final concentration of 0.02% in the medium. Incubation was continued for a further 3 h at 20°C with shaking (180 rpm) and the cells were then collected by centrifugation (15 min, 10,000 × *g*, 4°C) and frozen at $-<cps:it>20</cps:it{>}^{\circ }$C.

### Purification of Recombinant Enzyme CelRH5

Pellets obtained from 150 mL cultures were thawed and re-suspended in lysis buffer B-PER Bacterial Extraction Reagent (Thermo Scientific) supplemented with lysozyme and DNase I. Additionally cells disruption involved sonication on ice (five cycles for 60 s pulse and 30 s pause). Cell debris from the lysate was removed by centrifugation (20 min, 10,000 × *g*, 4°C) and the cell-free supernatant was diluted with equal volume of Equilibration Buffer (20 mM sodium phosphate, 300 mM sodium chloride, 10 mM imidazole; pH 7.4) and applied on re-equilibrated HisPur Ni-NTA Resin (Thermo Scientific) according to the manufacturer’s protocol. Unbound proteins were washed out using Wash Buffer (20 mM sodium phosphate, 300 mM sodium chloride, 25 mM imidazole; pH 7.4). Elution Buffer (20 mM sodium phosphate, 300 mM sodium chloride, 250 mM imidazole; pH 7.4) was used to extract His-tag bound proteins from the resin. Fractions were combined and dialyzed in 20 mM sodium phosphate buffer (pH 6.5) for 24 h at 10°C. The enzyme preparation was stored at 4°C for further analysis.

### Enzyme Assays

Recombinant *E. coli* TOP10/pBAD/*celRH5* clone able to produce metagenome-derived cellulase was used in activity assays on LB plates supplemented with L-arabinose (0.02% final concentration), carbenicillin (100 μg/mL final concentration) and various substrates (0.1% final concentration) such as soluble CMC, xylan from beech wood, arabic gum and insoluble Avicel (Sigma-Aldrich). Activity from recombinant clones was detected by staining plates, after incubation at 37°C for 24 h, with 1% (w/v) Congo red dye and de-stained with 1 M NaCl. Positive results were seen as a clear zone around the recombinant colonies. Clones were also tested with dyed substrates such as soluble OBR-HEC, Remazol Brilliant Blue-xylan (RBB-xylan) and insoluble AZCL-HEC, AZCL-xylan (Megazyme). Positive results were seen as a change in the ambient color of colonies. A negative control strain *E. coli* TOP10 transformed with plasmid pBAD/*Myc*HisA was used.

As a standard procedure to measure the recombinant enzyme CelRH5 activity, an assay with OBR-HEC solution as a substrate was performed (Biely et al., 1985; Ito et al., 2004). The reaction mixture contained 150 μL of 0.33% OBR-HEC in 20 mM sodium phosphate buffer (pH 6.5) and 50 μL of enzyme preparation. Incubation was carried out at 30°C for 30 min. These parameters were then taken as the standard conditions. The reaction was stopped with 600 μL of acetone and unprocessed substrate was removed by centrifugation for 5 min at 12,000 × *g*. The absorbance of the supernatant was measured at 550 nm. One unit (U) of cellulase activity was defined as the amount of enzyme required to release low molecular weight product containing 1 μmol of OBR from the dyed HEC per minute, under standard conditions.

The substrate specificity of the purified enzyme CelRH5 was determined at 30°C in 20 mM phosphate buffer (pH 6.5) using various substrates. The cellulase activity toward such polysaccharides as CMC, HEC, Avicel, corn stover (Sigma-Aldrich), soluble and insoluble chitin from crab shells (Sigma-Aldrich) and beech wood xylan was assayed by measuring the amount of reducing sugars released from polysaccharide using dinitrosalicylic acid (DNS) according to the Miller’s method and D-glucose was used as a standard (Miller, 1959). To measure the CelRH5 activity, reaction mixtures containing 100 μL of enzyme preparation and 400 μL of the appropriate substrate (1% final concentration) in 20 mM phosphate buffer (pH 6.5) was incubated at 30°C for 1 h. The reaction was terminated by mixing 100 μL of sample with 50 μL of DNS and by incubation at 90°C for 5 min. After that samples were cooled on ice and 500 μL of distilled water was added. The cellulase activity was assayed by measuring the increase in absorbance at 490 nm owing to the release of reducing sugars from the substrates. One unit of cellulase specific activity was defined as the amount of enzyme required to hydrolyze the substrate and to release 1 μmol of reducing sugars within 1 min per 1 mg of protein.

Beta-glucosidase, cellobiohydrolase, and β-xylosidase activities were determined using the respective chromogenic substrates *p*-nitrophenol-glucopyranoside (*p*NPG), *p*-nitrophenol-cellobioside (*p*NPC) and *p*-nitrophenol-xylopyranoside (*p*NPX) (Sigma-Aldrich) at 3 mM concentrations in 20 mM phosphate buffer (pH 6.5) were used. The activity of CelRH5 was assayed by measuring the increase in absorbance at 405 nm owing to the release of *p*-nitrophenol from the chromogenic substrates. One unit of enzyme activity was defined as the amount of enzyme liberating 1 μmol of *p*-nitrophenol from the substrate in 1 min under the standard reaction conditions.

### Protein Determination and Molecular Weight Estimation

The protein concentration in samples was determined according to the Bradford method using Coomassie Plus^TM^ Assay kit (Thermo Scientific) and with bovine serum albumin (BSA) as a standard (Walker, 2002). All fractions from the purification step were also separated using 12% polyacrylamide gel electrophoresis in denaturing conditions according to the Laemmli method (Walker, 2002) to establish the purity of the enzyme preparation and molecular weight of the enzyme monomer. The samples, with denaturing loading buffer, were incubated at 95°C for 10 min. Electrophoresis was performed for 150 min at 100 V at room temperature. To visualize proteins in the gel, staining with Coomassie Brilliant Blue was performed (Walker, 2002).

### Biochemical Characterization of CelRH5

The purified enzyme CelRH5 was used to determine the specificity and different biochemical properties under various conditions. Reactions were carried out under standard conditions with OBR-HEC as a substrate, and the reaction was stopped after 30 min with acetone. Cellulase activity was examined by measuring the absorbance of dyed low molecular weight product released from OBR-HEC, as described above, as the standard procedure. In all activity assays CelRH5 preparations contained 0.03 mg of enzyme per 1 mL. All experiments were conducted in triplicate.

The optimum pH was determined by assaying the CelRH5 enzyme activity with OBR-HEC dissolved in 20 mM Britton-Robinson buffer in pH values between 4.0 and 10.0 (with 0.5 pH unit gradations). Enzyme in appropriate pHs was incubated at 20°C for 30 min and the reactions were stopped with acetone. Activity of the enzyme was measured using the standard procedure. The highest activity obtained during assays was defined as the control, and defined as 100% activity. The pH stability profiles were determined by incubating the enzyme in 20 mM Britton-Robinson buffer solutions (pH 4.5–8.5, with 1.0-pH unit gradations) at 20°C up to 24 h with two checkpoints after 1 h and 7 h. Enzyme activity during incubation was established under standard conditions. Activity of the enzyme obtained at 0 h was defined as the control (100% activity).

The thermo-dependency of CelRH5 was assayed at various temperatures between 0 and 90°C (with 10°C gradations) by preparing samples as described in standard procedure and by incubation of the reaction mixture at appropriate temperatures for 30 min. The highest activity obtained during assay was defined as the control and was defined as 100% activity. The thermostability profiles were determined by incubating the enzyme at appropriate temperatures 4°C, 10°C, 20°C, 30°C, 40°C, and 50°C up to 54 h with two checkpoints after 2 h and 6 h. Enzyme activity during this experiment was determined by removing aliquots from the incubating sample and then by using the standard enzyme activity assays previously described.

The effect of various metal ions (Na^+^, K^+^ in 1, 10 and 100 mM concentrations, and Li^+^, Mn^2+^, Mg^2+^, Ca^2+^, Zn^2+^, Ni^2+^, Co^2+^, Fe^3+^ in 1 and 10 mM concentrations), final products of cellulose hydrolysis (glucose and cellobiose in 1, 10 and 100 mM concentrations) and other selected reagents (urea, guanidine HCl, DTT, glycine, SDS, 2-ME, DMSO, glycerol, ethanol and EDTA in various concentrations) (Figure 9C) on enzyme CelRH5 activity was determined by pre-incubating the enzyme with the individual reagents in 20 mM sodium phosphate buffer (pH 6.5) for 1 h followed by activity determination under standard conditions. Samples without metal ions and tested reagents were defined as controls representing 100% activity.

The kinetic parameters of freshly purified enzyme were established at 30°C using OBR-HEC as a substrate (in final concentrations of 0.75, 1.5, 3.0, 4.5, 6.0, and 7.5 mg/mL), diluted in 20 mM sodium phosphate buffer (pH 6.5). To determine Michaelis constant (*K*_m_), maximal velocity (*V*_max_) and catalytic constant (*k_cat_*) for the enzyme, CelRH5 reaction rate versus substrate concentration were plotted and calculated using Michaelis–Menten model with the GraphPad Prism 7.02 for Windows application.

#### Statistical Calculations

The arithmetic mean and the standard deviations were calculated. Simple classification variance analysis (ANOVA) tests were applied to determine significant differences. Firstly, the analysis of homogeneity of variance and normal distribution were performed by Hartley–Cochran–Bartlett and Kolmogorov–Smirnov tests, respectively. Subsequently ANOVAs were conducted to demonstrate the similarities or statistical differences between data. Finally, Tukey HSD tests was performed for the *post hoc* analyses. All statistical calculations were performed in STATISTICA 13.1.

## Results

### Metagenomic Library Construction and Screening Cellulase Clones

From 45,000 total clones of the TO-NF soil metagenomic library approximately 15,600 clones were screened for cellulolytic activity. One very strongly positive clone RH5_TO-NF021-E23 was observed and this was confirmed by re-plating the clone on LB agar plates with OBR-HEC where a strong zone of clearance was observed. Moreover, the cellulase activity was also observed after re-transformation of the isolated fosmid into fresh *E. coli* EPI300^TM^ cells. The fosmid from the RH5-TO-NF021-E23 clone, which contained an insert of 33,291 bp, was then sequenced and was found to contain 30 putative ORFs (Supplementary Figure 1A). The homology to other proteins and conserved domains were determined using the BLASTp and CDD applications, respectively (Supplementary Table 1), with ORF 23, 1,080 bp in length, showing similarity with endo-1,4-glucanases belonging to the GH5 family. The deduced product of gene *celRH5* consisted of 359 amino acid residues with a calculated molecular mass of 40.5 kDa and an isoelectric point of 7.7. The CelRH5 amino acid sequence of the metagenome-derived cellulase appears to be enclosed within one catalytic domain containing 268 amino acid residues and CelRH5 appears to contain a signal peptide of 31 amino acids (Supplementary Figure 1B). Analysis with SignalP-4.1 and Target P1.1 suggested that the cleavage site is likely to be located between position Ala31 and Gln32 (Emanuelsson et al., 2007).

**FIGURE 1:**
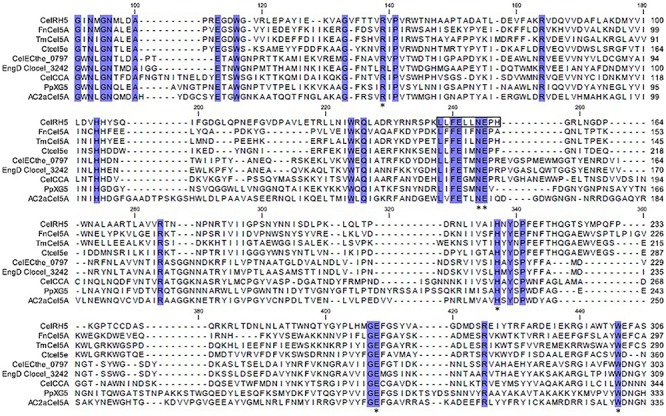
Multiple alignments of CelRH5 with the eight most homologous GH5 proteins deposited in the PDB database. Identical residues are shaded with a blue background. Fragment estimated with Inter ProScan as the catalytic site is marked with a black rectangle. The star symbol (^*^) indicates the conserved residues in the substrate-binding pocket as determined by 3D structure modeling.

However, no carbohydrate-binding module (CBM), which is often present in cellulose degrading enzymes, could be identified. Using ScanProsite the catalytic site appears to be present from aa 238 to 247 within the conserved signature sequence LLFELLNEPH with the conserved glutamic acid at position 245 (Figure 1).

**FIGURE 2:**
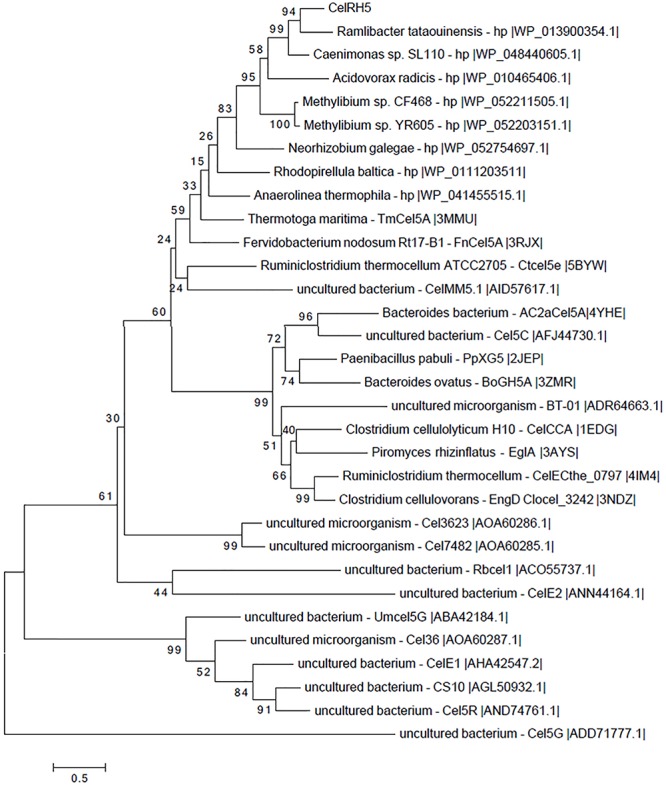
Phylogenetic tree of the CelRH5 amino acid sequence compared to closely related GH5 and other characterized metagenome-derived GH5. Analysis was performed using the MEGA 6.0 application. The tree was constructed using the Maximum Likelihood Method and the LG+G model with bootstrap analysis using 1,000 replicates.

Sequence analyses of the putative cellulase *celRH5* gene with BLASTn revealed no significant nucleotide similarity to known cellulases with only 66% and 68% relative identities being observed with putative endoglucanases from *Ramlibacter tataouinensis* 5–10 and *Ramlibacter tataouinensis* TTB310, respectively (data not shown). In addition, amino acid sequence analysis did not show very high homology with previously reported and characterized cellulases derived from cultivable microorganisms (Table 1). The most significant similarity with NCBI reference protein sequences was mainly exhibited by putative proteins with the highest hits of 65–66% of identity for *Ramlibacter tataouinensis* spp. (WP_061501559.1, WP_013900354.1) and *Caenimonas* sp. SL110 (WP_048440605.1). Moreover, analyses with BLASTp based on PDB were performed to determine the similarities to biochemically and structurally characterized cellulases. Results showed that CelRH5 displayed the highest similarity with the endo-β-1,4-endoglucanase TmCel5A from *Thermotoga maritima*, FnCel5A from *Flavidobacterium nodosum* Rt17B1, and Ctcel5e from *Ruminiclostridium thermocellum* ATCC 27405 with CelRH5, sharing 39%, 36%, and 34% identity, respectively (Chhabra et al., 2002; Mahadevan et al., 2008; Pereira et al., 2010; Wang et al., 2010; Yuan et al., 2015). Phylogenetic analysis of amino acid sequences obtained with MEGA 6.0 revealed that the CelRH5 cellulase had the highest homology to putative endoglucanases from the *Beta-proteobacteria* group belonging to the *Comamonadaceae* family (Figure 2). These results are in agreement with those obtained from the ORFs analyses on the metagenomic DNA fragment from the RH5_TO-NF021-E23 clone (Supplementary Table 1).

**TABLE 1:** Amino acid sequence CelRH5 homology to other sequences deposited in database established using BLASTp.

**Most homologous microorganism**	**Query cover**	**Identity**	**Accession no.**
*Ramlibacter tataouinensis*^a^	94%	65%	WP_061501559.1
*Ramlibacter tataouinensis*^a^	91%	66%	WP_013900354.1
*Caenimonas* sp. SL110 ^a^	83%	68%	WP_048440605.1
*Thermotoga maritima*^b^	93%	39%	3MMU, 3AZR, 3AMG, 3AOF
*Flavidobacterium nodosum* Rt17B1^b^	93%	36%	3RJX, 3RJX, 3NCO
*Ruminiclostridium thermocellum* ATCC 27405^b^	87%	32–34%	5BYW, 4U3A, 4U5I
*Ruminiclostridium thermocellum*^b^	95%	31%	4IM4
*Uncultured bacterium*^b^	61–62%	31%	4W84, 4W85, 4W86
*Bacillus licheniformis*^b^	86%	28%	4YZP
*Piromyces rhizinflatus*^b^	88%	26%	3AYR, 3AYS
*Paenibacillus pabuli*^b^	80%	25%	2JEP
*Bacteroides bacterium* AC2a^b^	93%	25%	4YHE, 4YHG
*Clostridium cellulovorans*^b^	94%	24%	3NDY, 3NDZ
*Clostridium cellulolyticum* H10^b^	92%	23%	1EDG
*Bacteroides ovatus*^b^	98%	22%	3ZMR

*^a^Sequences selected with BLASTp based on NCBI Protein Reference Sequences. ^b^Sequences selected with BLASTp based on PDB protein database and i-TASSER.*

### *In silico* Structural Analysis of CelRH5

**FIGURE 3:**
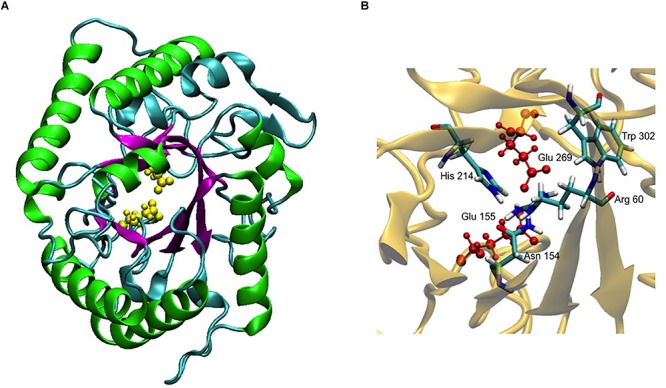
3D model of CelRH5. **(A)** TIM-barrel fold (β/α)_8_ present in GH5 (magenta and green) and the two catalytic Glu in yellow. **(B)** Conservative residues involved in catalysis.

**FIGURE 4:**
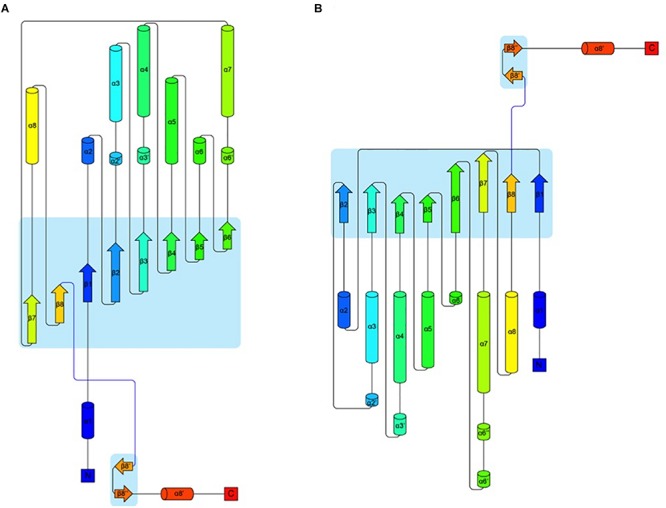
Topology of proteins CelRH5 **(A)** and 3MMU **(B)** showing the architecture of their monomers. The colouring is from blue at the N-terminus to red at the C-terminus. Parts of the chain that have a β-strand conformation are shown as arrows and α-helices are shown as cylinders (Stivala et al., 2011).

In an attempt to obtain a three-dimensional (3D) model of the CelRH5 protein, bacterial endo-β-1,4-glucanases were selected as templates by I-TASSER, with the following PDBs, all of which belong to GH5, identified as the top threading templates namely: 3MMU, 3AMD, and 3OAF from *Thermotoga maritima*; 1CEO from *Ruminiclostridium thermocellum*; 4YHE from *Bacteroidetes bacterium* AC2a together with 3NCO and 3RJY from *Fervidobacterium nodosum*. All the above cellulases belong to GH5 (Domínguez et al., 1996; Pereira et al., 2010; Wu et al., 2011; Zheng et al., 2012; Naas et al., 2015). A 3D model with TM-score of 0.73 ± 0.11 and C-score of 0.13 [the endoglucanase TmCel5A (PDB 3MMU) of *Thermotoga maritima*] was obtained from the first modeling round and was the best template. I-TASSER also identified the closest structural analogs in PDB (Table 2), all belonging to GH family 5. The TM-align values revealed that CelRH5 shows a high similarity with members of the GH5 family. Given that 3MMU was the best template found by I-TASSER and that 3RJX and 3MMU were found to be the two closest structural analogs in PDB, three additional modeling rounds using these PDBs were conducted in order to obtain a more accurate 3D model for CelRH5. The best 3D model was obtained using PDB 3MMU as a template with TM-score of 0.89 ± 0.09 and C-score of 0.58 (Figures 3A,B). The 3D model showed the common (β/α)_8_ TIM-barrel fold present in GH5 family members (Henrissat et al., 1995). In these modeling rounds, I-TASSER also identified endoglucanases belonging to GH family 5 as the closest structural neighbors for CelRH5. The topology of CelRH5 was found to be similar to the topology of PDB 3MMU (Figures 4A,B). In addition to the eight α-helices involved in the TIM-barrel architecture, CelRH5 possessed four additional short α-helices: α2′, α3′, α6′, and α10′ (Figure 4A). On the other hand, the predicted CelRH5’s 3D model revealed two additional β-strands on the C-terminal end of the protein (Figure 4A). Structural analogs, such as PDB 3MMU and 3RJY, also share these structural characteristics (Zheng et al., 2012).

**TABLE 2:** Top proteins identified as structural analogs in PDB.

**Rank**	**PDB**	**TM-score**	**RMSD**	**Cov.**	**Source**
1	3RJX	0.927	0.89	0.942	*Fervidobacterium nodosum*
2	3MMU	0.922	0.64	0.930	*Thermotoga maritima*
3	5BYW	0.911	1.26	0.936	*Ruminiclostridium thermocellum*
4	1EDG	0.873	2.42	0.948	*Clostridium cellulolyticum*
5	4YHE	0.832	2.23	0.945	*Bacteroidetes bacterium*
6	4X0V	0.870	2.35	0.942	*Caldicellulosiruptor* sp.
7	4IM4	0.869	2.04	0.927	*Ruminiclostridium thermocellum*
8	2JEP	0.868	2.04	0.930	*Paenibacillus pabuli*
9	3NDZ	0.868	2.12	0.927	*Clostridium cellulovorans*

*Ranking of proteins based on TM-score of the structural alignment between the query structure and known structures in the PDB. RMSD is the distance between residues that are structurally aligned by TM-align. ‘Cov.’ represents the coverage of the alignment by TM-align and is equal to the number of structurally aligned residues divided by length of the query protein.*

**FIGURE 5:**
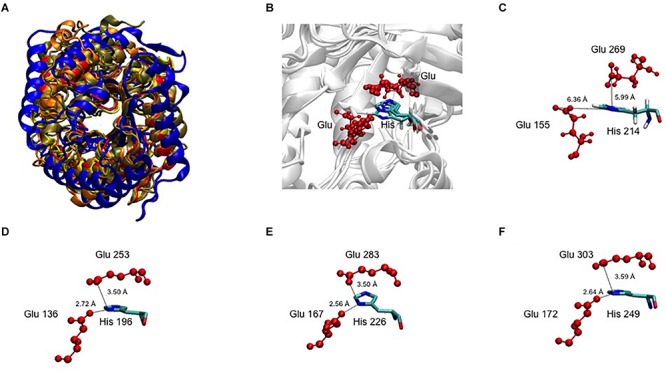
**(A)** The 3D superposition between CelRH5 and some structural neighbors. **(B)** The catalytic Glu and His 3D superposition between CelRH5 and some structural neighbors. **(C–F)** Evidence for the distance between the two catalytic Glu and His involved in the active center in CelRH5 and some structural neighbors 3MMU **(D)**, 3RXY **(E)** and 4YHE **(F)**.

A 3D superposition analysis between CelRH5 and some structural neighbors (PDBs 3MMU, 1CEO, 4YHE, 3NCO, and 3RJX) revealed a high structural homogeneity between the proteins (Figure 5A). The main differences in the overlapping proteins were found in the loop regions and not in the TIM-barrel fold. PDBs 3MMU and 3RJX showed the highest structural homogeneity with metric values for structural alignments (Q_H_-score) of 0.899 and 0.894, respectively, where the Q_H_-score for identical proteins is 1. Structural alignments with the GH analogs identified by I-TASSER allowed conserved residues known to be involved in the hydrolytic mechanism, in positions Glu155 (catalytic acid/base), Glu269 (nucleophile), Arg60, Asn154, His214, and Trp302 (Figure 3B), to be located in CelRH5. Two catalytic glutamates and the histidine which are essential for catalysis displayed the same spatial conformation when PDBs 3MMU, 4YHE, 3RJY, and the CelRH5 model was overlapped (Figure 5B). In CelRH5, the distances between the proton donor Glu155 to His214 and the nucleophile Glu269 to His214 are 6.36 and 5.99 Å, respectively (Figure 5C). These distances are higher than in others GH5 such as PDBs 3MMU (Figure 5D), 3RJX (Figure 5E), 4YHE (Figure 5F), *Cc*Cel5A and *Ph*Cel5G (Zheng et al., 2012).

### Cloning, Expression, and Purification of Recombinant *celRH5* Cellulase

**FIGURE 6:**
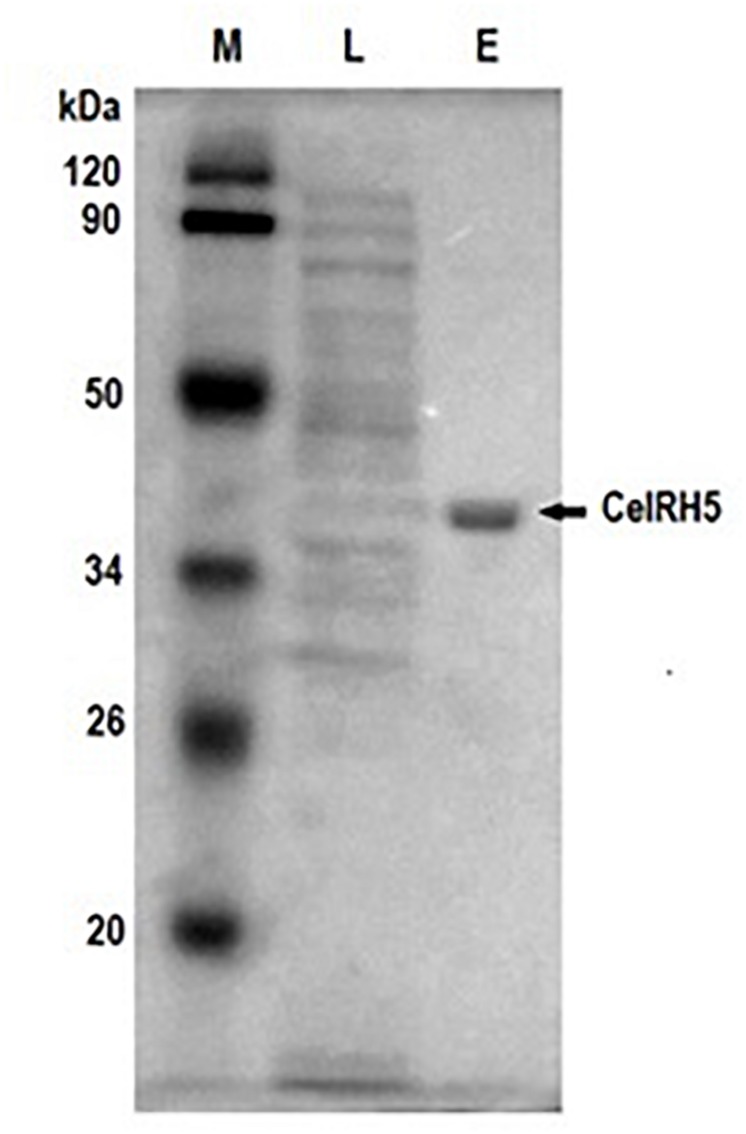
SDS-PAGE of the recombinant cellulase CelRH5 during purification steps. Lane M contains the protein molecular weight markers, lane L is the lysate from the *E. coli* TOP10/CelRH5 cells after protein overexpression, lane E is the recombinant enzyme CelRH5 eluted following the purification step with the His-tag Ni-NTA Resin.

In order to biochemically characterize CelRH5, it was necessary to sub-clone the gene and to heterologously express the enzyme in *E. coli*. To this end the *celRH5* gene together with a putative signal sequence was PCR amplified and cloned into the pBAD/*Myc*-HisA expression vector under arabinose inducible pBAD promoter, transformed into *E. coli* TOP10 cells and the transformants were tested for cellulase activity. The overexpressed His-tagged cellulase was subsequently purified using the one step nickel-immobilized metal affinity chromatography (Ni-IMAC) method under native conditions resulting in a purification fold of 18.53 (Table 3). The final yield of the recombinant CelRH5 cellulase produced was 3 mg pure protein with a specific activity of 5.56 U/mg per 1 L of recombinant *E. coli* TOP10/pBAD/*celRH5* culture. The recombinant enzyme was purified to homogeneity as determined by SDS-PAGE (Figure 6) and the molecular weight of the monomer was estimated to be approximately 40 kDa which corresponding to the expected molecular weight calculated from the CelRH5 amino acid sequence.

**TABLE 3:** Summary of purification of CelRH5 cellulase from 150 mL of *E. coli* TOP10/pBAD/*celRH5* culture.

**Purification step**	**Total protein (mg)**	**Total activity (U)**	**Specific activity (U/mg)**	**Purification fold**	**Yield (%)**
Lysate	18.78	5.67	0.30	1.00	100
Ni-IMAC	0.45	2.50	5.56	18.53	44

*Activity was performed using standard assay with OBR-HEC as a substrate. Protein determination was performed with Bradford method.*

### Biochemical Characterization of Recombinant Cellulase CelRH5

#### Effect of pH and Temperature on CelRH5 Activity

**FIGURE 7:**
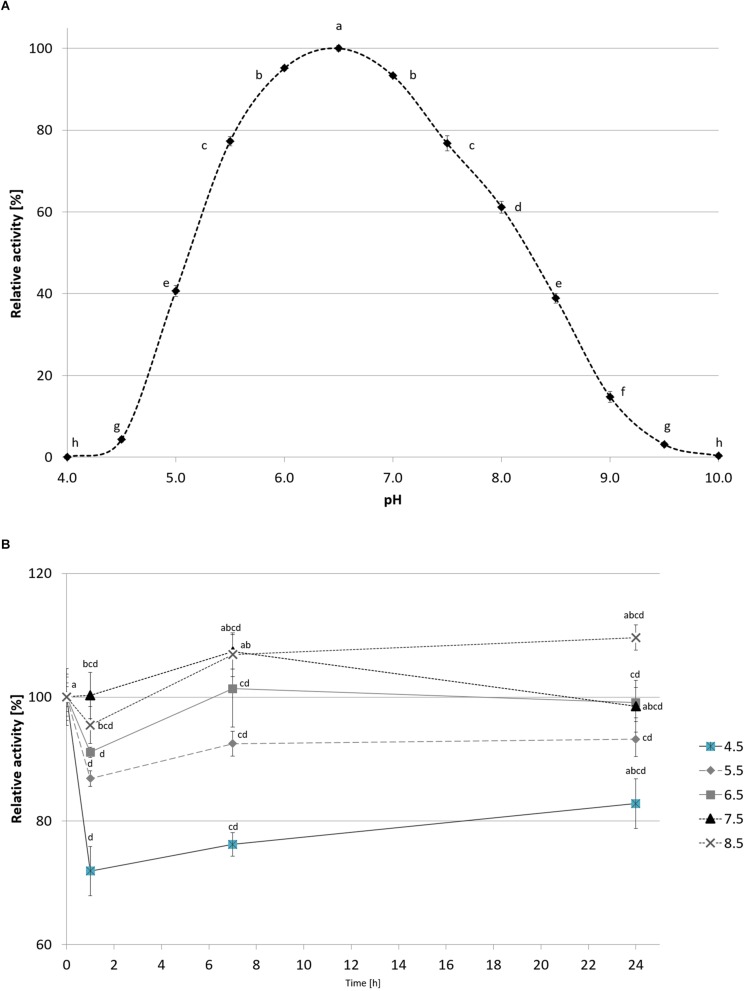
The effect of pH on recombinant CelRH5 activity **(A)** and stability **(B)**. Activity toward OBR-HEC was determined at 20 mM Britton-Robinson buffer at 20°C. The error bars represent the standard deviation (*n* = 3) and different letters mean statistically significant differences.

**FIGURE 8:**
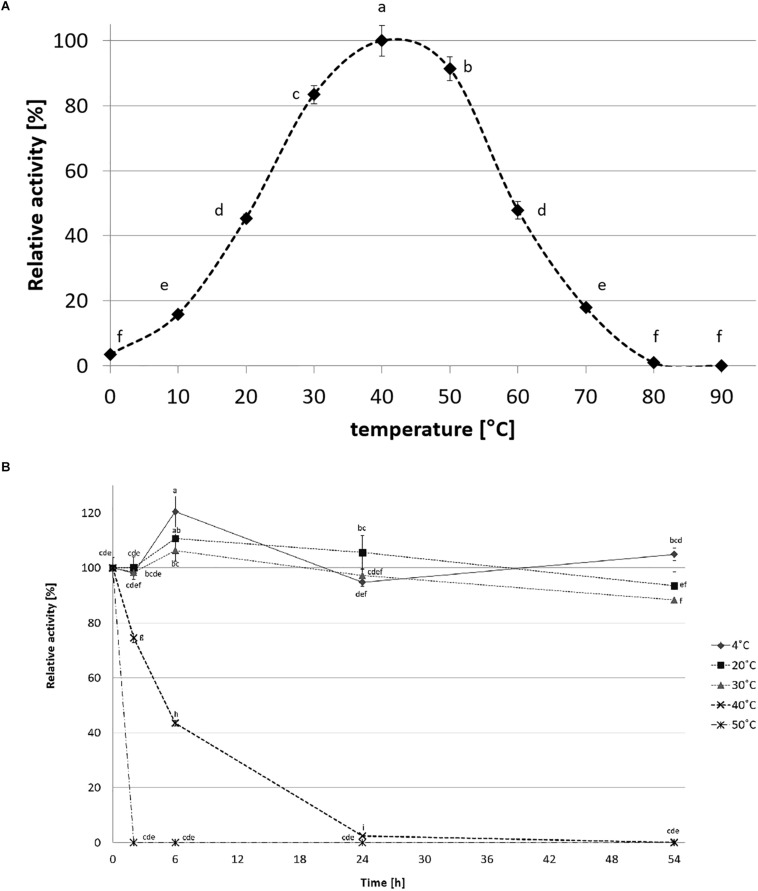
The effect of temperature on recombinant CelRH5 activity **(A)** and stability **(B)**. Activity toward OBR-HEC was determined at 20 mM sodium phosphate buffer pH 6.5. The error bars represent the standard deviation (*n* = 3) and different letters mean statistically significant differences.

CelRH5 activity was measured in the pH range 4.0–10.0. The recombinant enzyme exhibited the highest activity at pH 6.5, displayed almost 80% of its activity at pH 5.5–7.5 and remained stable at a range of pH 4.5–8.5 after incubation in 30°C for 24 h (Figure 7). The effect of temperature on CelRH5 activity was assessed in the temperatures range 0–90°C, with maximum activity being observed at 40°C, however, CelRH5 was stable up to 30°C after 54 h of incubation but above this temperature the relative activity decreased markedly. After 6 h of incubation at 40°C the enzyme exhibited about 40% relative activity, however, at 50°C after 2 h the activity was undetectable (Figure 8).

#### Substrate Specificity of CelRH5

The substrate specificity of the CelRH5 was determined in two ways. Initially the recombinant clone *E. coli* TOP10/pBAD/*celRH5* was qualitatively assayed on selective media supplemented with various substrates (data not shown). Results obtained from these qualitative screens indicated that the CelRH5 enzyme, when heterologously expressed in *E. coli*, was active only in the presence of soluble cellulose such as CMC, OBR-HEC and insoluble cellulose AZCL-HEC, with no activity being observed in media supplemented with beechwood xylan, RBB-xylan, AZCL-xylan, avicel or arabic gum. Subsequently the substrate specificity of the purified CelRH5 was quantitatively determined (Table 4). CelRH5 showed activity only with soluble polysaccharides such as CMC and HEC. In turn, no activity with other substrates such as Avicel, beechwood xylan, crab shells, soluble and insoluble chitin, corn stover or *p*-nitrophenol-glucopyranoside (*p*NPG), *p*-nitrophenol-cellobioside (*p*NPC), and *p*-nitrophenol- xylopyranoside (*p*NPX) was detected.

**TABLE 4:** Substrate specificity of recombinant CelRH5.

**Substrate**	**CelRH5 (U/mg)**
CMC	1,58 ± 0,03^a^
HEC	0,65 ± 0,05^a^
Avicel	0^a^
Beech wood xylan	0^a^
Crab shells chitin (soluble)	0^a^
Crab shells chitin (insoluble)	0^a^
Corn stover	0^a^
*p*-NPG	0^b^
*p*-NPC	0^b^
*p*-NPX	0^b^

*Specific activity was determined in optimal conditions for CelRH5 using ^a^Miller’s method or ^b^by quantification of released *p*-NP from chromogenic substrate; values represent the specific activity ± standard deviation (*n* = 3).*

#### The Effect of Metal Ions and Chemical Reagents on CelRH5 Activity

**FIGURE 9:**
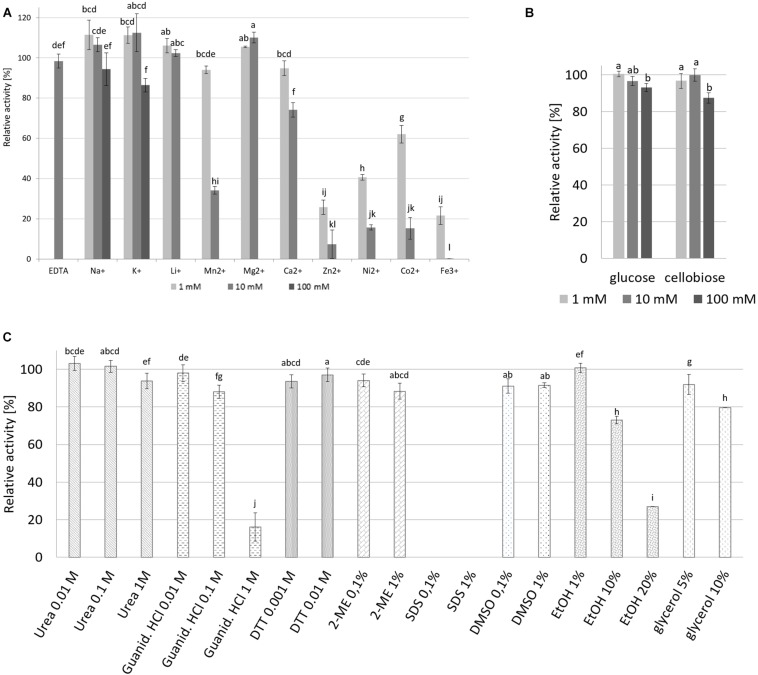
The effect of metals, sugars and various chemical agents on recombinant CelRH5 activity. **(A)** Influence of EDTA and metal ions on the CelRH5 activity. **(B)** Influence of glucose and cellobiose on the CelRH5 activity. **(C)** Influence of selected reagents on the CelRH5 activity. Control activity is sample without addition of reagents representing 100%. The error bars represent the standard deviation (*n* = 3) and different letters mean statistically significant differences.

The addition of metal ions such as Na^+^, K^+^ Li^+^, and Mg^2+^ at concentrations of 1 mM and 10 mM resulted in an increase in cellulase activity even up to 10% above the maximal activity, however, at higher concentrations addition of Na^+^ and K^+^ (100 mM) resulted in decreases in enzyme activity to 94% and 86%, respectively (Figure 9A). Decreases in cellulase activity were also observed upon addition of other metal ions even at low concentrations, with Zn^2+^, Ni^2+^, Co^2+^ lowering the relative activity below 20% and Fe^3+^ addition resulting in the total inhibition of enzyme activity; while the addition of 10 mM EDTA resulted in only a small decrease in cellulase activity to approximately 98% of maximal activity. The addition of glucose and cellobiose, final products of cellulose degradation, did not have a marked effect on CelRH5 activity, with the addition of cellobiose at a concentration of 100 mM resulting in a slight decrease in relative activity to 87% (Figure 9B).

The impact of various other reagents on CelRH5 activity was also assessed (Figure 9C). CelRH5 activity was not affected to any great extent upon addition of denaturing agents such as guanidine-HCl or urea at concentrations of 0.01 M and 0.1 M, however, when the concentration of guanidine-HCl was increased to 1 M a decrease in relative activity to 15% was observed. Similarly, the addition of reducing agents such as DTT and 2-ME had no marked effect on cellulase activity, nor did the addition of ethanol at 1% (v/v); however, increased ethanol concentrations of 10% (v/v) and 20% (v/v) resulted in decreases its relative activity of 27% and 73%, respectively (Figure 9C). The addition of DMSO also resulted in slight reductions in relative activity as did the addition of glycerol at concentration 5% (v/v) and 10% (v/v). However, of the reagents tested, only the addition of the ionic detergent SDS at concentrations of 0.1% (v/v) and 1% (v/v) resulted in the complete inactivation of CelRH5 activity.

#### Kinetic Parameters of the CelRH5 Enzyme

The kinetic parameters of the metagenome-derived cellulase, CelRH5, were determined in standard conditions (20 mM sodium phosphate buffer pH 6.5, 30°C) using various concentrations of OBR-HEC as a substrate. Calculations of the *K*_m_, *V*_max_, and *k_cat_* parameters were performed with the GraphPad Prism 7.02 application based on Michaelis–Menten model. The *K*_m_ was 0.675 mg/mL, while the *V*_max_ and *k_cat_* parameters were calculated as 14.27 μmol.min^–1^ per mg of protein and 9.75 s^–1^, respectively.

## Discussion

Function-based metagenomics involving the heterologous expression of environmental DNA without the need for culture isolation or sequence based analyses has led to the identification of numerous enzymes in metagenomic clone libraries (Nacke et al., 2012; Jackson et al., 2015; Borchert et al., 2017). Because of their industrial utility much interest has focused on the functional screening of metagenomics libraries for novel cellulases. A number of metagenome derived cellulases have been reported to date, from a variety of sources including animal rumens (Cheng et al., 2016); insects and nematodes feeding on cellulose (Zhang et al., 2013; Lee et al., 2014), and from a variety of other environments (Alvarez et al., 2013; Martin et al., 2014; Meneses et al., 2016; Yang et al., 2016), including soil (Berlemont et al., 2009; Zhou et al., 2016; Pimentel et al., 2017). Moreover, analyses based on metagenomic sequences revealed that soils are rich in glycosyl hydrolases which decompose plant polysaccharides including cellulose (Berlemont and Martiny, 2016). Here the functional screening of a rhizosphere-derived metagenomic clone library led to the identification of the *celRH5* gene with endoglucanase activity. Phylogenetic analysis indicated that the nucleotide sequence of the 1,080 bp *celRH5* gene was related to a putative endo-1,4-glucanase from *Ramlibacter tataouinensis* spp. showing 66–68% identity (Figure 2). BLASTp analysis of the deduced amino acid sequence indicated that CelRH5 exhibited the highest homology with hypothetical proteins from *Caenimonas* sp. SL110 and *Ramlibacter tataouinensis* spp. showing 66% and 68% of identity, respectively; with identity to other putative proteins being below 60%. With respect to previously characterized homologs of CelRH5 the highest identity was observed with thermostable cellulases TmCel5A from *Thermotoga maritima*, (Chhabra et al., 2002); FnCel5A from *Flavidobacterium nodosum* Rt17B1 (Wang et al., 2010) and Ctcel5e from *Ruminiclostridium thermocellum* ATCC 27405 (Yuan et al., 2015) with CelRH5 sharing 39%, 36%, and 34% identity, respectively, with those enzymes. The sequence identity between the deduced amino acid sequence of CelRH5 and other cellulase sequences in the databases are less than 70%, indicating that our *celRH5* gene is novel.

Bioinformatic analysis revealed that CelRH5 appears to possess one full catalytic domain composed of 268 aa typical of GH5, together with an N-terminal signal peptide composed of 31 aa. Moreover, the *in silico* generated 3D model indicated that CelRH5 exhibited the highest structural homology to other endo-β-1,4-glucanases from the GH5 family. Thus both the sequence analysis and the structural similarity suggest that CelRH5 belongs to the GH5 with endo-β-1,4-endoglucanase activity. GH5 (formerly known as “cellulase family A”) is one of the largest families of all glycosyl hydrolases (Aspeborg et al., 2012; Lombard et al., 2014). In general, GHs use acid–base catalysis to cleave glycosidic bonds with the hydrolytic mechanism in GH5 involving two strictly conserved glutamate residues; the catalytic acid/base and the nucleophile (Henrissat et al., 1995). In addition, a conserved histidine residue (His226 in PDB 3RJX) located in the substrate-binding site has been shown to be relevant for catalysis (Zheng et al., 2012). In addition, four other amino acids (Arg, Asn, His, Trp) located in the substrate-binding pocket are highly conserved in the GH5 family (Hilge et al., 1998). The amino acid and structural alignments using CelRH5 and their structural analog GHs identified by I-TASSER allowed us to locate these residues in CelRH5: Glu155 (catalytic acid/base) and Glu269 (nucleophile); and Arg60, Asn154, His214, and Trp302 (Figures 3, 5A,B). All of these residues were located in the canonical position previously reported in the closest structural neighbors PDBs 3MMU and 3RJX (Pereira et al., 2010; Zheng et al., 2012). The identification of these six strictly conserved residues in GH family 5 members provides additional evidence indicating that CelRH5 is a glycosyl hydrolase and belongs to family 5. Also, bioinformatic analyses revealed the presence of the catalytic triad (Glu–His–Glu) involving two glutamate residues and a histidine residue that are essential for catalytic activity and the same spatial conformation was observed when the CelRH5 model was superimposed on it’s nearest structural neighbors, *Tm*Cel5A (PDB 3MMU, *AC_2a_*Cel5A (PDB 4YHE) and *Fn*Cel5A (PDB 3RJX) (Figure 5B). However, while spatial conformation is important for catalytic efficiency, the distance between the Nδ1 atom of the imidazole group of histidine and the carboxyl groups of the two catalytic glutamates is also believed to play an important role in catalysis; with distances of 3.5 Å for the hydrogen atoms involved being optimal (Zheng et al., 2012). This structural aspect is interesting since it suggests that some GH5 enzymes may have evolved an electron relay network to facilitate more efficient catalysis (Zheng et al., 2012). However, in CelRH5 the distances between the proton donor Glu155 to His214 and the nucleophile Glu269 to His214 are greater than in other related GH5 members (Zheng et al., 2012), with the distance between Glu155 and Glu269 in CelRH5 being 3.56 Å. Thus, based on this CelRH5 may exhibit a lower activity than *Fn*Cel5A (PDB 3RJX), *Tm*Cel5A (PDB 3MMU), *AC_2a_*Cel5A (PDB 4YHE), *Cc*Cel5A and *Ph*Cel5G. The distance between Glu155 and Glu269 in CelRH5 is comparable with the distance between equivalent residues in other GH5 members (3.69 Å in 3RJX and 3.59 Å in 3MMU). Regardless, GH5 is one of the largest and well characterized GH families, and constitutes a group of monospecific enzymes as well as enzymes that demonstrate a large variety of specificities. Currently GH5 exemplifies a family which contains over 20 experimentally determined enzyme activities denoted with an EC number including endoglucanase, β-mannanase, exo-1,3-glucanase, endo-1,6-glucanase, xylanase and many others (Aspeborg et al., 2012; Lombard et al., 2014). With this in mind, biochemical assays were performed to further characterize the purified CelRH5 enzyme.

While functional metagenomics is a very promising approach in the isolation and identification of novel gene variants, problems can be encountered with expression in heterologous hosts; particularly with respect to the formation of inclusion bodies (Kennedy et al., 2008; Lambertz et al., 2014) Although our recombinant *celRH5* gene construct which we had cloned into the arabinose inducible expression vector pBAD/*Myc*-HisA contained a signal sequence and while activity was observed, high levels of the recombinant CelRH5 enzyme were present within the *E. coli* TOP10 cells indicating the likelihood of inclusion bodies being formed. We overcame this problem by inducing protein expression at the lower temperature of 20°C for a short 3-h time period, during which time the culture was shaking with a spiral coil placed in the medium which allowed additional aeration. This allowed the one step purification of the soluble CelRH5 enzyme using the Ni-IMAC method with a purification yield of 44% (Table 3 and Figure 6). The specific activity with OBR-HEC as substrate increased from 0.3 U/mg in the crude cell lysate to 5.56 U/mg after Ni-NTA affinity chromatography, which is an 18-fold purification of the recombinant enzyme; allowing 3 mg of purified CelRH5 enzyme to be obtained from a 1 L culture of *E. coli*.

We subsequently biochemically characterized the recombinant CelRH5 enzyme investigating the influence of temperature, pH, metal ions and chemical reagents on enzyme activity, together with substrate specificity. OBR-HEC was used as a substrate as it proved to be sensitive and stable under the different assay conditions. With respect to the influence of pH, CelRH5 shows approximately 80% of relative activity within the pH range between 5.5 and 7.5 with the highest activity at pH 6.5 (Figure 7). The enzyme also displayed good tolerance to a broad pH range (4.5–8.5), retaining a residual activity above 80% after 24 h of incubation at 30°C. Thus the endoglucanase CelRH5 is more active in slightly acidic conditions but is also quite active and stable under slightly alkaline conditions. While a number of other metagenome-derived endoglucanases from GH5 have also shown highest activity in pH range 4.5–7.0 (Feng et al., 2007; Alvarez et al., 2013; Martin et al., 2014; Lin et al., 2016), there are fewer examples of endoglucanases exhibiting activity over such a broad pH range (Lee et al., 2014; Garg et al., 2016; Lin et al., 2016).

CelRH5 has a temperature optimum of 40°C (Figure 8A). Thermostability experiments which involved incubating the enzyme at different temperatures for up to 54 h determined that CelRH5 is stable at temperatures up to 30°C. Incubation at higher temperatures such as 40°C for 2 h, 6 h, and 24 h decreased the relative activity to 73%, 42%, and 2%, respectively, while at 50°C the enzyme was inactive after 2 h (Figure 8B). Nonetheless CelRH5 is also active and stable at lower temperatures displaying 16% and 45% relative activity at 10°C and 20°C, respectively. However, despite the fact that bioinformatic analysis revealed that CelRH5 is most similar to the thermostable cellulases TmCel5A and FnCel5A with optimal temperature of approximately 80°C, and with Ctcel5e with an optimal temperature of 50°C; CelRH5 possesses thermal properties that are more typical of enzymes derived from psychrophilic microorganisms (Pereira et al., 2010; Wang et al., 2010). This property distinguishes CelRH5 from other previously characterized soil metagenome-derived GH5 cellulases (Voget et al., 2006; Alvarez et al., 2013; Garg et al., 2016; Pimentel et al., 2017), which exhibited activity and stability at higher temperatures even though they were isolated from metagenomic libraries of mesophilic or psychrophilic origin; with optimal activities between 40 and 55°C and stability ranging from 40°C and 60°C (Voget et al., 2006; Berlemont et al., 2009; Liu et al., 2010; Nacke et al., 2012). CelRH5 is similar biochemically with respect to pH and temperature optima to a halotolerant cold active marine GH5 endoglucanase, CelMM5 derived from the metagenome of the brown alga *Ascophyllum nodosum*. This enzyme also possesses a broad range of pH stability between 4.0 and 10.0 with highest activity at pH 7.0 and exhibits highest activity at 40°C, but is also stable up to 30°C (Martin et al., 2014).

The effects of various metal ions and reagents on CelRH5 activity was assessed with different effects being observed in the presence of various metal ions. Na^+^, K^+^, Li^+^, and Mg^2+^ at concentrations up to 10 mM resulted in slight increases in activity of up to 10%, whereas the addition of Zn^2+^, Ni^2+^, Co^2+^, and Fe^3+^ ions at the same concentrations resulted in decreases in relative activity to below 20% (Figure 9A). In addition when the chelating agent EDTA at 10 mM was added CelRH5 retained 98% of the relative activity. Similar results were obtained for cellulase C67-1 derived from buffalo rumen, belonging to the GH5 family, where EDTA had no impact on the enzyme activity, whereas ions of Zn^2+^, Cu^2+^, Cr^2+^, Mn^2+^, Co^2+^, and Fe^2+^ decreased its relative activity (Duan et al., 2009). These results suggest that CelRH5 is not a metallo-enzyme and its catalytic activity is not dependent on metal ions. In general CelRH5 appears to be quite a stable enzyme with no marked reduction in it’s activity being observed upon addition of a number of different reagents including reducing agents such as DTT and 2-ME, urea, guanidine-HCl (below 0.1 M), DMSO or glycerol (Figure 9C). Major reductions in the relative activity of CelRH5 were only observed upon addition of 1 M guanidine-HCl and ethanol at either 10 or 20%. In addition, the anionic detergent SDS completely inactivated CelRH5 even at low concentrations 0.1% (v/v) (Figure 9C), which has previously also been reported for other GH5 family cellulases (Lee et al., 2014; Martin et al., 2014; Garg et al., 2016; Lin et al., 2016; Pimentel et al., 2017). CelRH5 does not appear to be repressed by either cellobiose or by glucose which are the major end products of cellulose degradation, where at 100 mM concentrations only slight decreases in activity to 87% and 93%, respectively, were observed (Figure 9B). It is well established that the accumulation of glucose and cellobiose, the end products of hydrolysis, typically inhibit cellulases and decrease overall glucose yields (Hsieh et al., 2014). This substrate inhibition phenomenon is known as the “high solids effect” and negatively impacts on cellulose hydrolysis on an industrial scale and consequently on the commercial aspects of bioethanol production (Cannella and Jorgensen, 2014). Thus given the lack of inhibition observed in the presence of cellobiose and glucose then CelRH5 may be a useful enzyme to use in cellulase enzyme cocktails for bioethanol production.

Enzymes from GH5 family typically represent both monospecific and multi-specific enzymes which exhibit a range of diverse activities, including amongst others endo-β-1,4-glucanase, endo-β-1,4-xylanase, β-glucosidase, chitosanase, β-mannosidase activities (Aspeborg et al., 2012; Lombard et al., 2014). While the specificity of CelRH5 was examined with a broad range of substrates (Table 4) activity was only observed with CMC and hydroxyethylcellulose (HEC), with no activity being detected with Avicel, the crystalline form of cellulose. These substrates mainly contain β-1,4-glycosidic bonds but differ in solubility. The CelRH5 enzyme was also not active against aryl-glycosidic substrates such as *p*NPC, *p*NPG, and *p*NPX indicating that CelRH5 is a monospecific endo-β-1,4-glucanase that acts on internal *O*-glycosidic bonds in soluble homo-polysaccharides composed of glucose units. This is supported by our *in silico* structural analysis of the predicted CelRH5 3D model which strongly indicates that this enzyme should be classified as a new protein which belongs to GH5 family with endo-β-1,4-glucanase activity. The lack of CelRH5 activity toward an insoluble form of cellulose might be due to the apparent absence of a cellulose-binding module (CBM) in the enzyme’s predicted structure (Mahadevan et al., 2008; Wang et al., 2010, 2012; Reyes-Ortiz et al., 2013; Lee et al., 2014; Zhou et al., 2016), which was shown for other cellulases from the GH5 family, resulting in the ability to only degrade the amorphous form of cellulose (Voget et al., 2006; Mahadevan et al., 2008; Lee et al., 2014). The cellulases TmCel5A from *Thermotoga maritima* and FnCel5A from *Fervidobacterium nodosum*, which are the most related homologs of CelRH5, also contain only one catalytic GH5 family domain and while they display a spectrum of activity toward various substrates (Chhabra et al., 2002; Wang et al., 2010) they are not active against Avicel. TmCel5A has, however, been domain engineered, involving addition of a CBM domain to increase its activity toward Avicel by between 12- and 18-fold (Mahadevan et al., 2008). A similar CBM domain engineering approach with FnCel5A had a similar effect on the enzyme’s activity toward Avicel (Wang et al., 2012). Thus, affinity for the various forms of cellulose, amorphous and crystalline, may be related to the presence of additional CBMs covalently attached to the enzyme. It has been previously shown that many carbohydrate glycoside hydrolases contain additional CBMs in their structures that affect the ability to bind to insoluble substrates (Horn et al., 2012; Pakarinen et al., 2014). Such natural cellulolytic systems often combine several endo- and exo-acting enzymes with various preferences to different forms of the substrate. Their co-operation leads to the decomposition of crystalline and amorphous cellulose with glucose monomers being released (Pérez et al., 2002; Sukumaran et al., 2005; Voget et al., 2006; Fontes and Gilbert, 2010; Kuhad et al., 2011; Blumer-Schuette et al., 2012; Del Pozo et al., 2012; Horn et al., 2012; Lambertz et al., 2014; Pakarinen et al., 2014). Moreover, effective and complete decomposition of polysaccharides such as cellulose involve not only GH family enzymes but also many others, i.e., polysaccharide lyases, carbohydrate esterases or lytic polysaccharide monooxygenases (LPMOs). In the last 10 years groups of LPMOs, classified as Auxiliary Activity Family 9-11, 13-6, have been described, which generate optimal ends for endoglucanase acting enzymes and are common in bacteria, viruses and fungi (Horn et al., 2012; Lombard et al., 2014; Pakarinen et al., 2014; Filiatrault-Chastel et al., 2019). The endo-acting endoglucanases with LPMOs introduce new reducing or non-reducing chain ends for the exo-acting glucanases, which release cellobiose that is converted by β-glucosidases to glucose. Synergistic actions of these enzymes guarantees the efficient decomposition of lignocellulose biomass.

In summary, our results indicate that CelRH5 is a novel β-1,4-endoglucanase belonging to the GH5 family, which is adapted to low temperatures and which has a wide pH tolerance. The CelRH5 enzyme can be thermally inactivated in a short period of time by increasing the temperature to 50°C. The enzyme is also active in the presence of final cellulose degradation products, metal ions and various reagents, which are common in many technological processes indicating it’s potential suitability for industrial applications. Moreover, cold-adapted enzymes which are used in technological processes allow the use of temperature sensitive substrates and reagents, reducing overall energy consumption and costs. Also, enzymes which are active in low temperatures help to avoid contamination with mesophilic pathogens, which is very important in the food and animal feed industries. In addition, heterologously expressed cellulases, and in particular those that are stable at different pHs and which are active in presence of various chemicals, are desirable characteristics for cellulases in the chemical and detergent industries (Gomes and Steiner, 2004; Sukumaran et al., 2005; Li et al., 2009; Kuhad et al., 2011; Vester et al., 2014). It is also worth noting that cellulases which lack the CBM domain, as previously described (Pakarinen et al., 2014), are more easily recovered after hydrolysis and can be reused in subsequent rounds of cellulose processing. Thus, it is clear that CelRH5 may find utility in a variety of different biotechnological applications.

## Data Availability

The sequence of gene *celRH5* for these study can be found in GenBank under accession number MK640554.1 (https://www.ncbi.nlm.nih.gov/nuccore/MK640554.1). The sequence of metagenomic clone RH5_TO-NF021-E23 can be found in the GenBank under accession number MK640553.1 (https://www.ncbi.nlm.nih.gov/nuccore/MK640553).

## Author Contributions

AW-W, RH, JK, RB-G, and AD conceived and designed the experiments. AW-W, RH, and LM-Á performed the experiments. AW-W, RH, RB-G, and AD analyzed the data. SJ, JK, and AD contributed to the reagents, materials, and analysis tools. AW-W, RB-G, SJ, and AD wrote the manuscript.

## Conflict of Interest Statement

The authors declare that the research was conducted in the absence of any commercial or financial relationships that could be construed as a potential conflict of interest.

## Footnotes

1 https://www.reportbuyer.com/product/3392475

2 http://blast.ncbi.nlm.nih.gov/Blast.cgi

## References

[B1] Allgaier M. Reddy A. Park J. I. Ivanova N. D’haeseleer P. Lowry S. (2010). Targeted discovery of glycoside hydrolases from a switchgrass-adapted compost community. *PLoS One* 5:e8812. 10.1371/journal.pone.0008812 20098679 PMC2809096

[B2] Alvarez T. M. Paiva J. H. Ruiz D. M. Cairo J. P. L. F. Pereira I. O. Paixão D. A. A. (2013). Structure and function of a novel cellulase 5 from sugarcane soil metagenome. *PLoS One* 8:e83635. 10.1371/journal.pone.0083635 24358302 PMC3866126

[B3] Aspeborg H. Coutinho P. M. Wang Y. Brumer H. Henrissat B. (2012). Evolution, substrate specificity and subfamily classification of glycoside hydrolase family 5 (GH5). *BMC Evol. Biol.* 12:186 10.1186/1471-2148-12-186 PMC3526467 22992189

[B4] Bashir Y. Singh P. Konwar B. K. (2014). Metagenomics: an application based perspective. *Chin. J. Biol.* 2014:146030 10.1155/2014/146030

[B5] Berlemont R. Delsaute M. Pipers D. D’Amico S. Feller G. Galleni M. (2009). Insights into bacterial cellulose biosynthesis by functional metagenomics on Antarctic soil samples. *ISME J.* 3 1070–1081. 10.1038/ismej.2009.48 19458657

[B6] Berlemont R. Martiny A. C. (2016). Glycoside Hydrolases across environmental microbial communities. *PLoS Comput. Biol.* 12:e1005300. 10.1371/journal.pcbi.1005300 27992426 PMC5218504

[B7] Berman H. M. Westbrook J. Feng Z. Gilliland G. Bhat T. N. Weissig H. (2000). The protein data bank. *Nucleic Acids Res.* 28 235–242. 10.1093/nar/28.1.235 10592235 PMC102472

[B8] Biely P. Mislovièpvã D. Toman D. (1985). Soluble chromogenic substrates for the assay of endo-1,4-β-xylanases and endo-1,4-β-glucanases. *Anal. Biochem.* 144 142–146. 10.1016/0003-2697(85)90095-8 3838626

[B9] Blumer-Schuette S. E. Giannone R. J. Zurawski J. V. Ozdemir I. Ma Q. Yin Y. (2012). *Caldicellulosiruptor* core and pangenomes reveal determinants for noncellulosomal thermophilic deconstruction of plant biomass. *J. Bacteriol.* 194 4015–4028. 10.1128/jb.00266-12 22636774 PMC3416521

[B10] Borchert E. Selvin J. Kiran G. S. Jackson S. A. O’Gara F. Dobson A. D. W. (2017). A novel cold active esterase from a deep sea sponge *Stelletta normani* metagenomics library. *Front. Mar. Sci.* 4:287 10.3389/fmars.2017.00287

[B11] Cannella D. Jorgensen H. (2014). Do new cellulolytic enzyme preparations affect the industrial strategies for high solids lignocellulosic ethanol production? *Biotechnol. Bioeng.* 111 59–68. 10.1002/bit.25098 24022674

[B12] Cheng J. Huang S. Jiang H. Zhang Y. Li L. Wang J. (2016). Isolation and characterization of a non-specific endoglucanase from a metagenomic library of goat rumen. *World J. Microbiol. Biotechnol.* 32:12 10.1007/s11274-015-1957-4 26712627

[B13] Chhabra S. R. Shockley K. R. Ward D. E. Kelly R. M. (2002). Regulation of endo-acting glycosyl hydrolases in the hyperthermophilic bacterium *Thermotoga maritima* grown on glucan- and mannan-based polysaccharides. *Appl. Environ. Microbiol.* 68 545–554. 10.1128/aem.68.2.545-554.2002 11823189 PMC126696

[B14] De Castro E. Sigrist C. J. A. Gattiker A. Bulliard V. Langendijk-Genevaux P. S. Gasteiger E. (2006). ScanProsite: detection of PROSITE signature matches and ProRule-associated functional and structural residues in proteins. *Nucleic Acids Res.* 34 W362–W365. 10.1093/nar/gkl124 16845026 PMC1538847

[B15] Del Pozo M. V. Fernández-Arrojo L. Gil-Martínez J. Montesinos A. Chernikova T. N. Nechitaylo T. Y. (2012). Microbial β-glucosidase from cow rumen metagenome enhance the saccharification of lignocellulose in combination with commercial cellulase cocktail. *Biotechnol. Biofuels* 5:73 10.1186/1754-6834-5-73 PMC3477023 22998985

[B16] Domínguez R. Souchon H. Lascombe M. Alzari P. M. (1996). The crystal structure of a family 5 endoglucanase mutant in complexed and uncomplexed forms reveals an induced fit activation mechanism. *J. Mol. Biol.* 257 1042–1051. 10.1006/jmbi.1996.0222 8632467

[B17] Duan C. J. Xian L. Zhao G. C. Feng Y. Pang H. Bai X. L. (2009). Isolation and partial characterization of novel genes encoding acidic cellulases from metagenomes of buffalo rumens. *J. Appl. Microbiol.* 107 245–256. 10.1111/j.1365-2672.2009.04202.x 19302301

[B18] Emanuelsson O. Brunak S. von Heijne G. Nielsen H. (2007). Locating proteins in the cell using TargetP, SignalP and related tools. *Nat. Protoc.* 2 953–971. 10.1038/nprot.2007.131 17446895

[B19] Feng Y. Duan C. J. Pang H. Mo X. C. Wu C. F. Yu Y. (2007). Cloning and identification of novel cellulase genes from uncultured microorganisms in rabbit cecum and characterization of the expressed cellulases. *Appl. Microbiol. Biotechnol.* 75 319–328. 10.1007/s00253-006-0820-9 17216439

[B20] Ferrer M. Ghazi A. Beloqui A. Vieites J. M. López-Cortés N. Marín-Navarro J. (2012). Functional metagenomics unveils a multifunctional glycosyl hydrolase from the family 43 catalysing the breakdown of plant polymers in the calf rumen. *PLoS One* 7:e38134 10.1371/journal.pone.0038134 PMC3382598 22761666

[B21] Filiatrault-Chastel C. Navarro D. Haon M. Grisel S. Herpoël-Gimbert I. Chevret D. (2019). AA16, a new lytic polysaccharide monooxygenase family identified in fungal secretomes. *Biotechnol. Biofuels* 12:55 10.1186/s13068-019-1394-y PMC6420742 30923563

[B22] Fontes C. M. Gilbert H. J. (2010). Cellulosomes: highly efficient nanomachines designed to deconstruct plant cell wall complex carbohydrates. *Annu. Rev. Biochem.* 79 655–681. 10.1146/annurev-biochem-091208-085603 20373916

[B23] Garg R. Srivastava R. Brahma V. Verma L. Karthikeyan S. Sahni G. (2016). Biochemical and structural characterization of a novel halotolerant cellulase from soil metagenome. *Sci. Rep.* 6:39634 10.1038/srep39634 PMC5180356 28008971

[B24] Gasteiger E. Hoogland C. Gattiker A. Duvaud S. Wilkins M. R. Appel R. D. (2005). “Protein identification and analysis tools on the ExPasy server,” in *The Proteomics and Protocols Handbook*, ed. Walker J. M. (New York, NY: Humana Press), 571–607. 10.1385/1-59259-890-0:571

[B25] Gomes J. Steiner W. (2004). The biocatalytic potential of extremophiles and extremozymes. *Food Technol. Biotechnol.* 42 223–235.

[B26] Graham J. E. Clark M. E. Nadler D. C. Huffer S. Chokhawala H. A. Rowland S. E. (2011). Identification and characterization of a multidomain hyperthermophilic cellulase from an archaeal enrichment. *Nat. Commun.* 2:375 10.1038/ncomms1373 21730956

[B27] Henrissat B. Callebaud I. Fabrega S. Lehn P. Mornon J.-P. Davies G. (1995). Conserved catalytic machinery and the prediction of a common fold for several families of glycosyl hydrolases. *Proc. Natl. Acad. Sci. U.S.A.* 92 7090–7094. 10.1073/pnas.92.15.7090 7624375 PMC41477

[B28] Hilge M. Gloor S. M. Rypniewski W. Sauer O. Heightman T. D. Zimmermann W. (1998). High-resolution native and complex structures of thermostable b-mannanase from *Thermomonospora fusca* – substrate specificity in glycosyl hydrolase family 5. *Structure* 6 1433–1444. 10.1016/s0969-2126(98)00142-7 9817845

[B29] Horn S. J. Vaaje-Kolstad G. Westereng B. Eijsink V. G. H. (2012). Novel enzymes for the degradation of cellulose. *Biotechnol. Biofuels* 5:45 10.1186/1754-6834-5-45 PMC3492096 22747961

[B30] Hsieh C. C. Cannella D. Jorgensen H. Felby C. Thygesen L. G. (2014). Cellulase inhibition by high concentrations of monosaccharides. *J. Agric. Food Chem.* 62 3800–3805. 10.1021/jf5012962 24724847

[B31] Humphrey W. Dalke A. Schulten K. (1996). VMD - visual molecular dynamics. *J. Mol. Graph.* 14 33–38. 10.1016/0263-7855(96)00018-5 8744570

[B32] Ito F. Amano Y. Nozaki K. Saxena M. I Brown M. R. Jr. Kanda T. (2004). Hydrolysis of water-soluble and water- insoluble cellulosic substrates by endo-β-1,4-glucanase from *Acetobacter xylinum*. *J. Appl. Glycosci.* 51 297–301. 10.5458/jag.51.297

[B33] Jackson S. A. Borchert E. O’Gara F. Dobson A. D. W. (2015). Metagenomics for the discovery of novel biosurfactants of environmental interest from marine ecosystems. *Curr. Opin. Biotechnol.* 33 176–182. 10.1016/j.copbio.2015.03.004 25812477

[B34] Kennedy J. Marchesi J. R. Dobson A. D. W. (2008). Marine metagenomics: strategies for the discovery of novel enzymes with biotechnological applications from marine environments. *Microb. Cell Fact.* 7:27 10.1186/1475-2859-7-27 PMC2538500 18717988

[B35] Kuhad R. C. Gupta R. Singh A. (2011). Microbial cellulases and their industrial application. *Enzyme Res.* 2011:280696 10.4061/2011/280696 PMC3168787 21912738

[B36] Lambertz C. Garvey M. Klinger J. Heesel D. Klose H. Fischer R. (2014). Challenges and advances in the heterologous expression of cellulolytic enzymes: a review. *Biotechnol. Biofuels* 7:135 10.1186/s13068-014-0135-5 PMC4212100 25356086

[B37] Lee C. M. Lee Y. S. Seo S. H. Yoon S. H. Kim S. J. Hahn B. S. (2014). Screening and characterization of a novel cellulase gene from the gut microflora of *Hermetia illucens* using metagenomic library. *J. Microbiol. Biotechnol.* 24 1196–1206. 10.4014/jmb.1405.05001 25022521

[B38] Li L. L. McCorkle S. R. Monchy S. Taghavi S. van der Lelie D. (2009). Bioprospecting metagenomes: glycosyl hydrolases for converting biomass. *Biotechnol. Biofuels* 2:10 10.1186/1754-6834-2-10 PMC2694162 19450243

[B39] Li L. L. Taghavi S. McCorkle S. M. Zhang Y. B. Blewitt M. G. Brunecky R. (2011). Bioprospecting metagenomics of decaying wood: mining for new glycoside hydrolases. *Biotechnol. Biofuels* 4:23 10.1186/1754-6834-4-23 PMC3171299 21816041

[B40] Li W. Cowley A. Uludag M. Gur T. McWilliam H. Squizzato S. (2015). The EMBL-EBI bioinformatics web and programmatic tools framework. *Nucleic Acids Res.* 43 W580–W584. 10.1093/nar/gkv279 25845596 PMC4489272

[B41] Lin L. Liu X. Zhou Y. Guan L. He J. Huang W. (2016). A novel pH-stable, endoglucanase (JqCel5A) isolated from a salt-lake microorganism, *Jonesia quinghaiensis*. *Electron. J. Biotechnol.* 24 56–62. 10.1016/j.ejbt.2016.09.004

[B42] Liu J. Liu W. Zhao X. Shen W. Cao H. Cui Z. (2010). Cloning and functional characterization of a novel endo-β-1,4-glucanase gene from a soil-derived metagenomic library. *Appl. Microbiol. Biotechnol.* 89 1083–1092. 10.1007/s00253-010-2828-4 20938774

[B43] Lombard V. Golaconda Ramulu H. Drula E. Coutinho P. M. Henrissat B. (2014). The Carbohydrate-active enzymes database (CAZy) in 2013. *Nucleic Acids Res.* 42 D490–D495. 10.1093/nar/gkt1178 24270786 PMC3965031

[B44] Mahadevan S. A. Wi S. G. Lee D. S. Bae H. J. (2008). Site-directed mutagenesis and CBM engineering of Cel5A (*Thermotoga maritima*). *FEMS Microbiol. Lett.* 287 205–211. 10.1111/j.1574-6968.2008.01324.x 18752623

[B45] Marchler-Bauer A. Derbyshire M. K. Gonzales N. R. Lu S. Chitsaz F. Geer L. Y. (2015). CDD: NCBI’s conserved domain database. *Nucleic Acids Res.* 43 222–226. 10.1093/nar/gku1221 PMC4383992 25414356

[B46] Martin M. Biver S. Steels S. Barbeyron T. Jam M. Portetelle D. (2014). Identification and characterization of a halotolerant, cold active marine endo-β-1,4-glucanase by using functional metagenomics of seaweed-associated microbiota. *Appl. Environ. Microbiol.* 80 4958–4967. 10.1128/AEM.01194-14 24907332 PMC4135742

[B47] McWilliam H. Li W. Uludag M. Squizzato S. Park Y. M. Buso N. (2013). Analysis tool web services from the EMBL-EBI. *Nucleic Acids Res.* 41 W597–W600. 10.1093/nar/gkt376 23671338 PMC3692137

[B48] Mendes R. Garbeva P. Raaijmakers J. M. (2013). The rhizosphere microbiome: significance of plant beneficial, plant pathogenic, and human pathogenic microorganisms. *FEMS Microbiol. Rev.* 37 634–663. 10.1111/1574-6976.12028 23790204

[B49] Meneses C. Silva B. Medeiros B. Serrato R. Johnston-Monje D. (2016). A metagenomic advance for the cloning and characterization of a cellulase from red rice crop residues. *Molecules* 21:E831 10.3390/molecules21070831 PMC6274478 27347917

[B50] Miller G. L. (1959). Use of dinitro-salicylic acid reagent for determination of reducing sugars. *Anal. Chem.* 31 426–428. 10.1021/ac60147a030

[B51] Mori T. Kamei I. Hirai H. Kondo R. (2014). Identification of novel glycosyl hydrolases with cellulytic activity against crystalline cellulose from metagenomic libraries constructed from bacterial enrichment cultures. *Springerplus* 3:365 10.1186/2193-1801-3-365 PMC4112031 25077068

[B52] Naas A. E. MacKenzie A. K. Dalhus B. Eijsink V. G. H. Pope P. B. (2015). Structural features of a bacteroidetes-affiliated cellulase linked with a polysaccharide utilization locus. *Sci. Rep.* 5:11666 10.1038/srep11666 PMC4488959 26133573

[B53] Nacke H. Engelhaupt M. Brady S. Fischer C. Tautzt J. Daniel R. (2012). Identification and characterization of novel cellulolytic and hemicellulolytic genes and enzymes derived from German grassland soil metagenomes. *Biotechnol. Lett.* 34 663–675. 10.1007/s10529-011-0830-2 22187078 PMC3298741

[B54] Noguchi H. Park J. Takagi T. (2006). MetaGene: prokaryotic gene finding from environmental genome shotgun sequences. *Nucleic Acids Res.* 34 5623–5630. 10.1093/nar/gkl723 17028096 PMC1636498

[B55] O’Mahony M. M. Henneberger R. Selvin J. Kennedy J. Doohan F. Marchesi J. R. (2015). Inhibition of the growth of *Bacillus subtilis* DSM10 by a newly discovered antibacterial protein from the soil metagenome. *Bioengineered* 6 89–98. 10.1080/21655979.2015.1018493 25692994 PMC4601227

[B56] Pakarinen A. Haven M. Ø. Djajadi D. T. Várnai A. Puranen T. Viikari L. (2014). Cellulases without carbohydrate-binding modules in high consistency ethanol production process. *Biotechnol. Biofuels* 7:27 10.1186/1754-6834-7-27 PMC3974600 24559384

[B57] Pereira J. H. Chen Z. McAndrew R. Sapra R. Chhabra S. R. Sale K. L. (2010). Biochemical characterization and crystal structure of endoglucanase Cel5A from the hyperthermophilic *Thermotoga maritima*. *J. Struct. Biol.* 172 372–379. 10.1016/j.jsb.2010.06.018 20599513

[B58] Pérez J. Muñoz-Dorado J. de la Rubia T. Martßnez J. (2002). Biodegradation and biological treatments of cellulose, hemicellulose and lignin: an overview. *Int. Microbiol.* 5 53–63. 10.1007/s10123-002-0062-3 12180781

[B59] Petersen T. N. Brunak S. von Heijne G. Nielsen H. (2011). SignalP 4.0: discriminating signal peptides from transmembrane regions. *Nat. Methods* 8 785–786. 10.1038/nmeth.1701 21959131

[B60] Pimentel A. C. Ematsu G. C. G. Liberato M. V. Paixão D. A. A. Franco Cairo J. P. L. Mandelli F. (2017). Biochemical and biophysical properties of a metagenome-derived GH5 endoglucanase displaying an unconventional domain architecture. *Int. J. Biol. Macromol.* 99 384–393. 10.1016/j.ijbiomac.2017.02.075 28238914

[B61] Reyes-Ortiz V. Heins R. A. Cheng G. Kim E. Y. Elandt R. B. (2013). Addition of a carbohydrate-binding module enhances cellulase penetration into cellulose substrates. *Biotechnol. Biofuels* 6:93 10.1186/1754-6834-6-93 PMC3716932 23819686

[B62] Rice P. Longden I. Bleasby A. (2000). EMBOSS: the european molecular biology open software suite. *Trends Genet.* 16 276–277. 10.1016/s0168-9525(00)02024-2 10827456

[B63] Roy A. Kucukural A. Zhang Y. (2010). I-TASSER: a unified platform for automated protein structure and function prediction. *Nat. Protoc.* 5 725–738. 10.1038/nprot.2010.5 20360767 PMC2849174

[B64] Ryu S. Karim M. N. (2011). A whole cell biocatalyst for cellulosic ethanol production from dilute acid-treated corn stover hydrolysates. *Appl. Microbiol. Biotechnol.* 91 529–542. 10.1007/s00253-011-3261-z 21519935

[B65] Sharma A. Tewari R. Rana S. S. Soni R. Soni S. K. (2016). Cellulases: classification, methods of determination and industrial applications. *Appl. Biochem. Biotechnol.* 179 1346–1380. 10.1007/s12010-016-2070-3 27068832

[B66] Stivala A. Wybrow M. Wirth A. Whisstock J. C. Stuckey P. J. (2011). Automatic generation of protein structure cartoons with Pro-origami. *Bioinformatics* 27 3315–3316. 10.1093/bioinformatics/btr575 21994221

[B67] Sukumaran R. K. Singhania R. R. Pandey A. (2005). Microbial cellulases – production, applications and challenges. *J. Sci. Ind. Res.* 64 832–844.

[B68] Tamura K. Stecher G. Peterson D. Filipski A. Kumar S. (2013). MEGA6: Molecular evolutionary genetics analysis version 6.0. *Mol. Biol. Evol.* 30 2725–2729. 10.1093/molbev/mst197 24132122 PMC3840312

[B69] Tiwari R. Nain L. Labrou N. E. Shukla P. (2018). Bioprospecting of functional cellulases from metagenome for second generation biofuel production: a review. *Crit. Rev. Microbiol.* 44 244–257. 10.1080/1040841X.2017.1337713 28609211

[B70] Vester J. K. Glaring M. A. Stougaard P. (2014). Discovery of novel enzymes with industrial potential from a cold and alkaline environment by a combination of functional metagenomics and culturing. *Microb. Cell Fact.* 13:72 10.1186/1475-2859-13-72 PMC4035831 24886068

[B71] Voget S. Steele H. L. Streit W. R. (2006). Characterization of a metagenome-derived halotolerant cellulose. *J. Biotechnol.* 126 26–36. 10.1016/j.jbiotec.2006.02.011 16584799

[B72] Walker J. M. (2002). *The Protein Protocols Handbook*, 2nd Edn Totowa, NJ: Humana Press.

[B73] Wang Y. Tang R. Tao J. Wang X. Zheng B. Feng Y. (2012). Chimeric cellulase matrix for investigating intramolecular synergism between non-hydrolytic disruptive functions of carbohydrate-binding modules and catalytic hydrolysis. *J. Biol. Chem.* 287 29568–26878. 10.1074/jbc.M111.320358 22778256 PMC3436196

[B74] Wang Y. Wang X. Tang R. Yu S. Zheng B. Feng Y. (2010). A novel thermostable cellulase from *Fervidobacterium nodosum*. *J. Mol. Catal. B Enzym.* 66 294–301. 10.1016/j.molcatb.2010.06.006

[B75] Wu T. H. Huang C. H. Ko T. P. Lai H. L. Ma Y. Chen C. C. (2011). Diverse substrate recognition mechanism revealed by *Thermotoga maritima* Cel5A structures in complex with cellotetraose, cellobiose and mannotriose. *Biochim. Biophys. Acta* 1814 1832–1840. 10.1016/j.bbapap.2011.07.020 21839861

[B76] Xing M. N. Zhang X. Z. Huang H. (2012). Application of metagenomic techniques in mining enzymes from microbial communities for biofuel synthesis. *Biotechnol. Adv.* 30 920–929. 10.1016/j.biotechadv.2012.01.021 22306331

[B77] Yang C. Xia Y. Qu H. Li A. D. Liu R. Wang Y. (2016). Discovery of new cellulases from the metagenome by a metagenomics-guided strategy. *Biotechnol. Biofuels* 9:138 10.1186/s13068-016-0557-3 PMC4932676 27382415

[B78] Yuan S. F. Wu T. H. Lee H. L. Hsieh H. Y. Lin W. L. Yang B. (2015). Biochemical characterization and structural analysis of a bifunctional cellulase/xylanase from *Clostridium thermocellum*. *J. Biol. Chem.* 290 5739–5748. 10.1074/jbc.M114.604454 25575592 PMC4342484

[B79] Zhang C. Kim S. K. (2010). Research and application of marine microbial enzymes: status and prospects. *Mar. Drugs* 8 1920–1934. 10.3390/md8061920 20631875 PMC2901830

[B80] Zhang L. Fan Y. Zheng H. Du F. Zhang K. Huang X. (2013). Isolation and characterization of a novel endoglucanase from a *Bursaphelenchus xylophilus* metagenomic library. *PLoS One* 8:e82437 10.1371/journal.pone.0082437 PMC3873927 24386096

[B81] Zheng B. Yang W. Zhao X. Wang Y. Lou Z. Rao Z. (2012). Crystal Structure of hyperthermophilic endo-β-1,4-glucanase. *J. Biol. Chem.* 287 8336–8346. 10.1074/jbc.M111.266346 22128157 PMC3318711

[B82] Zhou Y. Wang X. Wei W. Xu J. Wang W. Xie Z. (2016). A novel efficient β-glucanase from a paddy soil microbial metagenome with versatile activities. *Biotechnol. Biofuels* 9:36 10.1186/s13068-016-0449-6 PMC4752780 26877766

