# Supplementary figures and images for: Biochemical Characterization of a Novel Monospecific Endo-β-1,4-Glucanase Belonging to GH Family 5 From a Rhizosphere Metagenomic Library
Source: Front Microbiol. 2019 Jun 14;10:1342. doi: 10.3389/fmicb.2019.01342 (PMC6587912; doi:10.3389/fmicb.2019.01342)

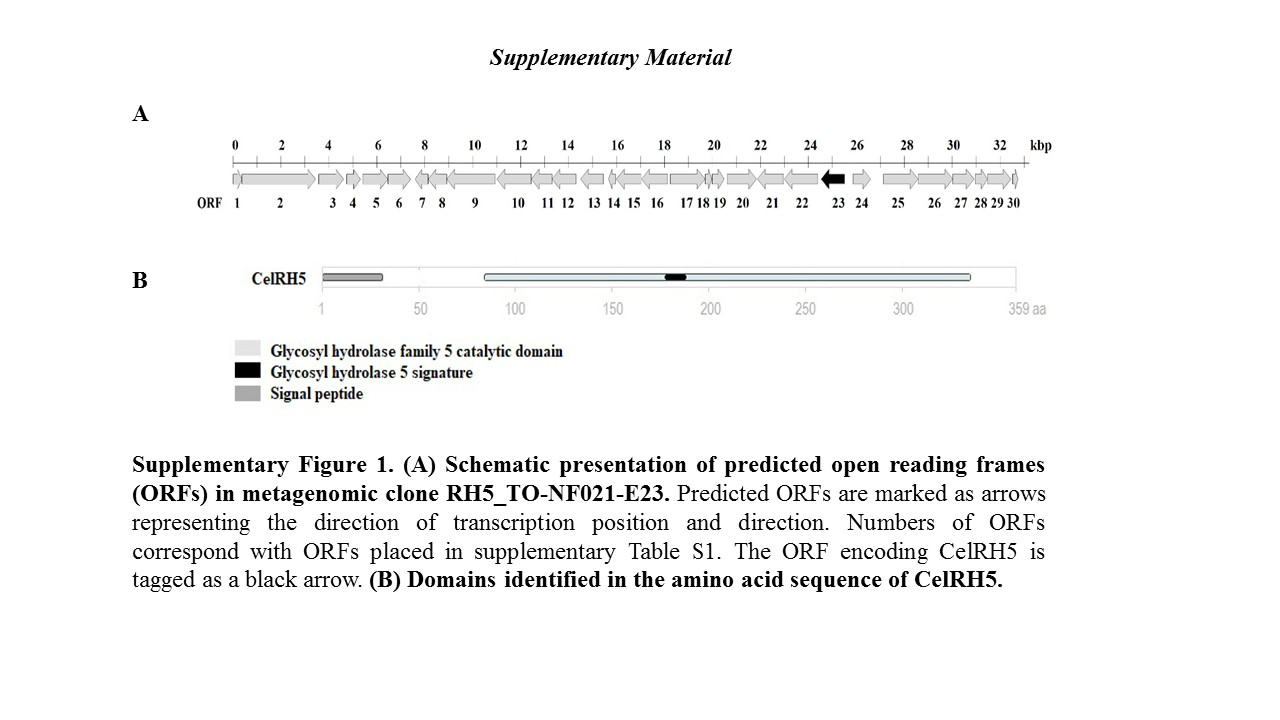

Supplement: Supplementary file 1 [file Image_1.TIF]
