# Supplementary material for: Biochemical Characterization of a Novel Monospecific Endo-β-1,4-Glucanase Belonging to GH Family 5 From a Rhizosphere Metagenomic Library
Source: Front Microbiol. 2019 Jun 14;10:1342. doi: 10.3389/fmicb.2019.01342 (PMC6587912; doi:10.3389/fmicb.2019.01342)
Supplement: Supplementary file 2 [file Table_1.DOCX]

Supplementary Material

**Supplementary Table 1.** **Presumable ORFs in metagenomic DNA fragment from clone RH5_TO-NF021-E23 corresponding to ORFs demonstrated on supplementary Figure S1.**

| **ORF** | **Strand** | **Length [aa]** | **Domain hit** | **Putative proteins** | **Coverage/ Identity [%]** | **E value** | **Accession No.** |
| --- | --- | --- | --- | --- | --- | --- | --- |
| 1* | + | 123 | RND_mfp | Hypothetical protein  [*Rhodoferax saidenbachensis*] | 81/77 | 1e-46 | WP_051391831.1 |
| 2 | + | 1026 | MMPL superfa, AcrB | Acriflavin resistance protein B [*Rhodoferax saidenbachensis*] | 99/87 | 0.0 | WP_029707226.1 |
| 3 | + | 329 | Aldo_ket_red, Tas | Aldo/keto reductase  [*Acidovorax* sp. KKS102] | 100/76 | 4e-179 | WP_015016169.1 |
| 4 | + | 191 | PRK15041 | Methyl-accepting chemotaxis protein  [*Hylemonella gracilis* ATCC 19624] | 100/58 | 3e-64 | EGI77005.1 |
| 5 | + | 357 | DEDDh, PRK09182 | DNA polymerase III subunit epsilon  [*Polaromonas* sp. CF318] | 96/59 | 6e-136 | WP_007868862.1 |
| 6 | + | 256 | HBDH_SDR_c, PRK12429 | 3-hydroxybutyrate dehydrogenase  [*Methylibium* sp. CF468] | 100/80 | 1e-148 | WP_047512437.1 |
| 7 | - | 170 | Not identified | Hypothetical protein  [*Rhodoferax saidenbachensis*] | 98/62 | 9e-71 | WP_029706284.1 |
| 8 | - | 269 | Exosortase_EpsH | Exosortase  [*Variovorax* sp.URHB0020] | 96/63 | 4e-97 | WP_028249828.1 |
| 9 | - | 698 | vWFA, VIT, LPP20, Marine_srt_targ | Vault protein inter-alpha-trypsin  [*Polaromonas naphthalenivorans*] | 91/69 | 0.0 | WP_011799464.1 |
| 10 | - | 486 | HATPase_c, HisKA, PRK11100 | Histidine kinase  [*Rhodoferax saidenbachensis*] | 98/66 | 0.0 | WP_029706290.1 |
| 11 | - | 249 | REC, trans_reg_c, PRK11083 | DNA-binding response regulator [*Variovorax* sp. URHB002] | 96/73 | 6e-117 | WP_028249823.1 |
| 12 | - | 383 | ATT_like, PRK09082 | Aminotransferase  [*Curvibacter gracilis*] | 99/85 | 0.0 | WP_027475288.1 |
| 13 | - | 313 | PBP2_CysB_like, HTH_1, PRK12682 | Transcriptional regulator  [*Caenimonas* sp. SL110] | 100/89 | 0.0 | WP_048441038.1 |
| 14 | - | 123 | CbiX_SirB_N | Cobalamin biosynthesis protein CbiX  [*Acidovorax oryzae*] | 96/61 | 2e-45 | WP_035225128.1 |
| 15 | - | 368 | LptG_IptG | LPS exprort ABC transporter permease LptG  [*Acidovorax* sp. NO-1] | 100/72 | 0.0 | WP_008904846.1 |
| 16 | - | 368 | LptF_YjgP | LPS export ABC transporter LptF  [*Ramlibacter tataouinensis*] | 98/65 | 2e-169 | WP_041675314.1 |
| 17 | + | 505 | Peptidase_M17, PRK00913 | Cytosol aminopeptidase  [*Rhodoferax saidenbachensis*] | 94/75 | 0.0 | WP_029709552.1 |
| 18 | + | 144 | DNA_pol3_chi | DNA polymerase III subunit chi [*Rhodoferax ferrireducens*] | 99/63 | 3e-58 | WP_011464409.1 |
| 19 | + | 105 | Not identified | Hypothetical protein  [*Acidovorax* sp.MR-S7] | 96/45 | 8e-23 | WP_020228078.1 |
| 20 | + | 375 | PBP1_ABC_LIVBP_like, Peripla_BP_6 | ABC transporter substrate-binding protein  [*Polaromonas* sp. JS666] | 100/91 | 0.0 | WP_011483328.1 |
| 21 | - | 367 | NTP_transferase, LbH_G1P_TT_C_like, GCD1 | Hypothetical protein Csp A03840  [*Curvibacter* putative symbiont of *Hydra magnipapillata*] | 100/80 | 0.0 | CBA27749.1 |
| 22 | - | 440 | UDPG_MGDP_dh_N, UDPG_ MGDP_dh, UDPG_ MGDP_dh_C, Ugd | UDP-glucose 6-dehydrogenase [*Curvibacter* putative symbiont of *Hydra magnipapillata*] | 100/87 | 0.0 | CBA27701.1 |
| **23** | **-** | **359** | **Cellulase** | **Endo-1,4-glucanase, Glycoside Hydrolase Family 5**  **[*Ramlibacter tataouinensis*]** | **91/66** | **1e-160** | **WP_013900354.1** |
| 24 | + | 297 | PRK08162 | Hypothetical protein  [*Leptolyngbya* sp. PCC6406] | 95/34 | 1e-49 | WP_008312556.1 |
| 25 | + | 496 | MATE_tuaB_like | Polysaccharide biosynthesis protein  [*Polaromonas glacialis*] | 98/50 | 4e-150 | WP_029524315.1 |
| 26 | + | 469 | Wzy_C, RfaL | Polymerase  [*Polaromonas glacialis*] | 88/39 | 4e-88 | WP_029524314.1 |
| 27 | + | 319 | Glyco_tranf_GTA_type, WcaA | Glycosyl transferase  [*Polaromonas glacialis*] | 98/50 | 5e-92 | WP_051675706.1 |
| 28 | + | 175 | LbH_SAT, CysE | Serine acetyltransferase  [*Derxia gummosa*] | 98/64 | 1e-73 | WP_028311934.1 |
| 29 | + | 348 | Succinoglycan_BP_ExoA, Gluco_tranf_2_3 | Glycosyl transferase  [*Polaromonas* sp. CF318] | 95/54 | 7e-119 | EJL83843.1 |
| 30* | + | 107 | Not identified | Hypothetical protein  [*Ideonella* sp. B508-1] | 96/61 | 8e-13 | WP_051048932.1 |

* - Partial ORFs; *celRH5* ORF is showed in bold letters
